# Peak Filtering, Peak Annotation, and Wildcard Search for Glycoproteomics

**DOI:** 10.1074/mcp.RA120.002260

**Published:** 2020-12-08

**Authors:** Abhishek Roushan, Gary M. Wilson, Doron Kletter, K. Ilker Sen, Wilfred Tang, Yong J. Kil, Eric Carlson, Marshall Bern

**Affiliations:** Research and Development Group, Protein Metrics Inc, Cupertino, California, USA

**Keywords:** Byonic, glycosylation, prenylation, neuropeptide, proSAAS, IsoTaG, FcγRIIIa, CD16a, ETD, electron transfer dissociation, FDR, false discovery rate, HCD, higher-energy C-trap dissociation, HUPO HGI, Human Proteome Organization Glycoproteomics Initiative, MS, mass spectrometry, PTMs, posttranslational modifications, PEP, posterior error probability, PSM, peptide-spectrum match, QTOF, quadrupole time-of-flight, XIC, extracted ion chromatogram

## Abstract

Glycopeptides in peptide or digested protein samples pose a number of analytical and bioinformatics challenges beyond those posed by unmodified peptides or peptides with smaller posttranslational modifications. Exact structural elucidation of glycans is generally beyond the capability of a single mass spectrometry experiment, so a reasonable level of identification for tandem mass spectrometry, taken by several glycopeptide software tools, is that of peptide sequence and glycan composition, meaning the number of monosaccharides of each distinct mass, *e.g.*, HexNAc(2)Hex(5) rather than man5. Even at this level, however, glycopeptide analysis poses challenges: finding glycopeptide spectra when they are a tiny fraction of the total spectra; assigning spectra with unanticipated glycans, not in the initial glycan database; and finding, scoring, and labeling diagnostic peaks in tandem mass spectra. Here, we discuss recent improvements to Byonic, a glycoproteomics search program, that address these three issues. Byonic now supports filtering spectra by *m/z* peaks, so that the user can limit attention to spectra with diagnostic peaks, *e.g.*, at least two out of three of 204.087 for HexNAc, 274.092 for NeuAc (with water loss), and 366.139 for HexNAc-Hex, all within a set mass tolerance, *e.g.*, ± 0.01 Da. Also, new is glycan “wildcard” search, which allows an unspecified mass within a user-set mass range to be applied to N- or O-linked glycans and enables assignment of spectra with unanticipated glycans. Finally, the next release of Byonic supports user-specified peak annotations from user-defined posttranslational modifications. We demonstrate the utility of these new software features by finding previously unrecognized glycopeptides in publicly available data, including glycosylated neuropeptides from rat brain.

## Highlights

• Filtering mass spectra by key peaks facilitates glycoproteomics search.
• Glycan wildcard search finds unanticipated N-linked glycans.
• User-defined diagnostic peaks for chemical biology.
• Low-abundance O-linked glycosylation on neuropeptides derived from ProSAAS.

## Graphical Abstract

**Figure:**
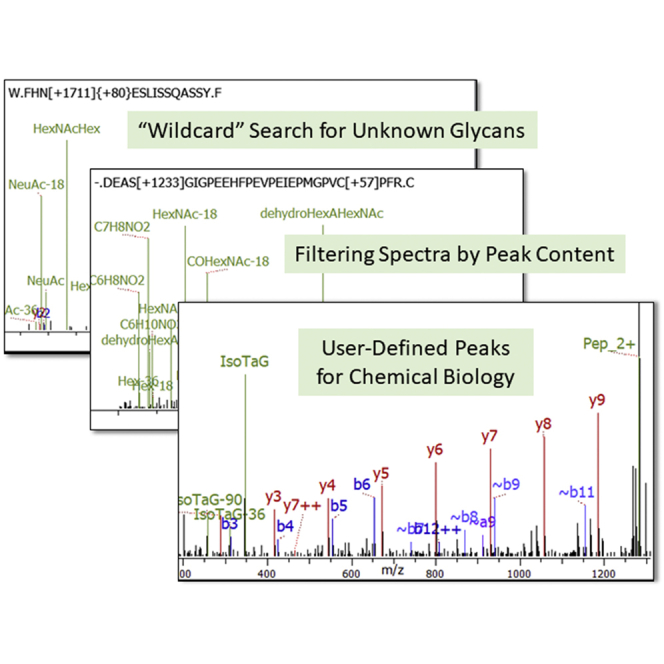

## In Brief

New features have been added to popular commercial software. The features are demonstrated on several publicly available data sets and are used to discover sulfated N-glycans on Fc gamma receptor IIIa, O-linked glycosylation on rat neuropeptides, and prenylated cysteines in human cells.

Site-specific glycosylation analysis (1) typically employs both single and tandem mass spectrometry (MS) to analyze digested glycoprotein samples at the level of intact glycopeptides. If the digestion separates glycosylation sites into distinct peptides, then the glycopeptide level gives site-specific information, but multiple specific digests or protease cocktails such as pronase (2) or chromatographic separation may be required to distinguish glycosylation on sites that do not separate with standard trypsin digestion. Hybrid approaches (3) augment glycopeptide analysis with intact glycoprotein analysis or “dissection” into large peptides; these approaches can detect correlations between remote glycosylation sites (4) and also offer rapid comparison across samples or conditions. Exoglycosidases (5) or specialized derivitizations (6) can be added to the MS methods to obtain more detailed information about structure and linkages.

Byonic is a commercial proteomics search engine that includes special support for glycopeptide analysis; it can be used as a node in Thermo Fisher’s Proteome Discoverer platform, as a component in Protein Metrics’ Byos workflows or as standalone software with a freeware viewer to look at results. Byonic has been bought by over 200 academic laboratories and 100 biopharmaceutical companies. Many if not most of these groups use Byonic for both “ordinary” proteomics and glycoproteomics, and the software is finding current use in coronavirus studies (7, 8, 9, 10, 11, 12). Byonic was the most popular search program in the recent Human Proteome Organization Glycoproteomics Initiative (HUPO HGI) Study, used by 12 out of 22 submissions, including 10 of the 13 submissions from groups that do not develop their own software (personal communication with Morten Thaysen-Andersen). Byonic assigns glycopeptide spectra by generating peptides *via in silico* digestion from a protein database and combining them with all glycan compositions from one or more glycan composition databases. Byonic then predicts a theoretical glycopeptide spectrum and compares it with each observed spectrum that has a precursor mass within a user-defined precursor mass tolerance. This approach is the logical extension of the approach taken by the most popular proteomics search tools such as Sequest (13), Mascot (14), and MaxQuant (15). Other software packages adopting database search for glycoproteomics include Protein Prospector (16, 17), GlycReSoft (18), I-GPA (19), and pGlyco (20).

The database-search approach to glycopeptide identification is quite successful in practice, especially for samples with overall glycosylation already characterized from glycomics experiments, scientific literature, or biological knowledge. Database search, however, cannot identify a glycopeptide with a glycan not in the glycan database. In extreme cases, a search might return no glycopeptide matches at all because of an inaccurate glycan database. Searching with a very large glycan database to minimize missed glycopeptides incurs another problem: false or partially false matches, *e.g.*, a peptide correct at only one terminus along with a glycan with extra or missing monosaccharides. For unusual or unnatural glycopeptide samples, such as those produced by chemical biology methods, even if database search returns correct glycopeptide matches, spectrum scoring and annotation may not accurately model the peaks found in the mass spectra, thereby compromising sensitivity and slowing manual validation. Here, we describe four new software features in Byonic that help address these issues:

1) **Inclusion List.** Controls for limiting attention to tandem mass spectra (denoted MS2 or MS/MS) within a user-defined elution time and m/z range;
2) **MS2 Peak Filtering.** Controls for limiting attention to tandem mass spectra containing user-defined or menu-selected *m/z* peaks;
3) **Custom Peaks.** User-defined scoring and labels for *m/z* peaks associated with posttranslational modifications;
4) **Glycan Wildcard Search.** Wildcard or “blind” modifications applied on top of glycan modifications.

Feature (1), Inclusion List, enables the user to run searches targeting MS2 spectra from specific precursors; for example, time slices where glycopeptides have been previously found or where co-eluting “stripes” appear in a heatmap view of a chromatogram. This feature is especially helpful for thorough iterative analysis of simple samples, *e.g.*, biopharmaceutical formulations.

Feature (2), MS2 Peak Filtering, similar to MS-Filter in Protein Prospector can be used to focus attention on glycopeptide spectra by limiting the search to spectra containing glycan oxonium ions at *m/z*’s such as 138.055, 204.087, and/or 274.092. As shown previously by Medzihradszky *et al.* (21), ordinary and especially phospho-proteomics data sets often contain unrecognized glycopeptide spectra that can be found by filtering for oxonium ions, and it is quite feasible to enrich for both phosphorylation and glycosylation simultaneously (22). Filtering by MS2 peaks can also be used to find all glycopeptides with the same peptide part by filtering for glycan-loss peaks such as those from the bare peptide (Y0) or the bare peptide plus HexNAc (Y1) (23). MS2 Peak Filtering provides a relatively quick way to test whether a chemical or metabolic labeling succeeded or to check a large data set for glycosylation or rare posttranslational modifications (PTMs) that produce reliable diagnostic peaks in MS2 with beam-type (quadrupole time-of-flight [QTOF] or higher-energy C-trap dissociation [HCD]) collisional dissociation. Examples include phosphotyrosine (24) and phospho-O-GlcNAc (25), as well as glycan monosaccharides such as phosphomannose and acetyl-NeuAc.

The Byonic Viewer, which is free software for viewing Byonic search results, already labels many diagnostic peaks for PTMs, including the peaks for the modifications just named, but new feature (3), Custom Peaks, gives the user a way to score and add labels to peaks not included in Byonic’s list of known diagnostic peaks. This feature should be especially useful for unusual samples such as bacterial glycopeptides and unnatural samples such as those produced by chemical biology.

Finally, glycan wildcard search offers a partial solution to the problem of choosing a glycan database. With a glycan wildcard with mass ranging from −300 to +300 Da, Byonic will be able to make assignments of the form GLTT[+HexNAc(1)Hex(1)NeuAc(2) + 291.092]PR, if the glycan database contains only HexNAc(1)Hex(1)NeuAc(2) and not HexNAc(1)Hex(1)NeuAc(3). The wildcard glycan is readily correctible to HexNAc(1)Hex(1)NeuAc(3), based on close agreement of the 291.092 wildcard mass with the true mass of 291.095 for NeuAc. Glycan wildcard search is quite different from allowing a large-mass wildcard, say one with range up to +1300 Da, on any serine or threonine. Depending upon the size of the glycan database and the wildcard mass range, glycan wildcard search may be several times faster, especially for N-linked glycosylation. And, more importantly, it allows Byonic to score spectra more accurately, because Byonic will predict peaks for oxonium ions (138, 204, 274, 292, 366, 657, etc.) and neutral losses of NeuAc, NeuAc(1)Hex(1), etc., for a modification of +HexNAc(1)Hex(1)NeuAc(2) + 291.092, but not for an “ordinary” wildcard modification of +1238.415, which is modeled as a nonfragmenting mass delta. Notice that a neutral loss of NeuAc(1)Hex(1) does not make sense for all possible topologies of HexNAc(1)Hex(1)NeuAc(2), but predicting a few extraneous peaks does not have a large impact on Byonic’s score, whereas not predicting highly reliable peaks such as 204 and 274 changes the score dramatically, because the lack of these peaks incurs large score penalties.

In the computational experiments described below, we aimed to demonstrate and test search speedup, as well as discovery of unsuspected glycopeptides in ordinary proteomics samples, from MS2 Peak Filtering; improved scoring and spectrum annotation from Custom Peaks; and unanticipated glycan matching, which leads to glycan database improvement or customization, from Glycan Wildcard Search.

Features (1), (2), and (4) appear in the June, 2020 release of Byonic (version 3.9.4). Feature (3), Custom Peaks, will appear in the September, 2020 release.

## Experimental Procedures

### Data Sets

We used the following publicly available proteomics data sets to test the software improvements:

1. Rat brain peptidomics data (26), ProteomeXchange PXD002431, especially file 20100805_Velos3_AnSe_Batch12faste_hypot_S3.raw. These “Copenhagen data” are from endogenous peptides extracted and separated with nLC and analyzed with HCD fragmentation and high-resolution MS2 on a Thermo LTQ Orbitrap Velos. The ProteomeXchange accession lists *Rattus rattus* (black rat), but the journal article names Sprague-Dawley, which is a *Rattus norvegicus* (brown rat) strain.
2. Rat brain peptidomics data (27), ftp://massive.ucsd.edu/MSV000080106. These “Wisconsin data” are Q-Exactive Orbitrap spectra from a study comparing neuropeptides in fed and unfed Sprague-Dawley rats. We used the files 041213_HT_A2.raw, 041213_HT_A3.raw, 041213_HT_A5.raw, 041213_HT_A6.raw, 041213_HT_B4.raw, and 041213_HT_B5.raw_20200705_Byonic.
3. Human T cells cultured with isotope-targeted glycoproteomics labels (IsoTaG), producing heavy and light O-linked N-azidoacetylGlcNAc in place of O-linked GlcNAc, ProteomeXchange PXD004559 (28). IsoTaG is a metabolic labeling method that allows enrichment with biotin and produces recognizable pairs of peaks in MS1, which are then targeted for MS2 analysis. The mass spectra are from a Thermo Orbitrap Elite and include HCD Orbitrap MS2 and CID and ETD ion-trap MS2. We used a single .raw file, 341_iso_glycan_trypsin_scout.raw.
4. Human endothelial cells (EA.hy92619) with prenylation probes YnF or YnGG (for farnesylation and geranylgeranylation, respectively) captured with three different azide reagents (29). The mass spectra in ProteomeXchange PXD009155 are from a Q-Exactive Orbitrap. We used six .raw files, JMS133D.raw to JMS133I.raw, along with the corresponding .mgf files.
5. Purified CD16a/FcγRIIIa protein (FCG3A_HUMAN, UniprotKB P08637) from human NK cells (30), ProteomeXchange PXD014127. The mass spectra are from a Q-Exactive Orbitrap. We used a single .raw file, NK13_CD16_glycopeptide.raw.
6. Human plasma enriched for glycoproteins from the HUPO HGI study. We used B_glycopepnew_HCDEThcDiTCIDpeptide.raw. The data are available from https://www.hupo.org/HPP-News/6272119

### Software

We added two new tabs to Byonic’s input UI, containing the controls for Inclusion List and MS2 Peak Filtering. The Inclusion List UI allows any number of elution time and m/z boxes, defined by “m/z begin”, “m/z end”, “Elution time begin”, and “Elution time end”; these can be set individually or imported from a spreadsheet (.csv file). The *m/z* limits apply to the monoisotopic *m/z*, which may be assigned either by the vendor software or by Byonic’s own precursor-calling code, depending upon the “Precursor and charge assignments” settings on the Advanced tab. (For targeted MS2, the “Originally assigned” precursor *m/z* is usually the center of the isolation window.) In any case, it is best to use an *m/z* window that is at least 2 Thomsons wide to account for monoisotopic *m/z* errors.

**Fig. 1:**
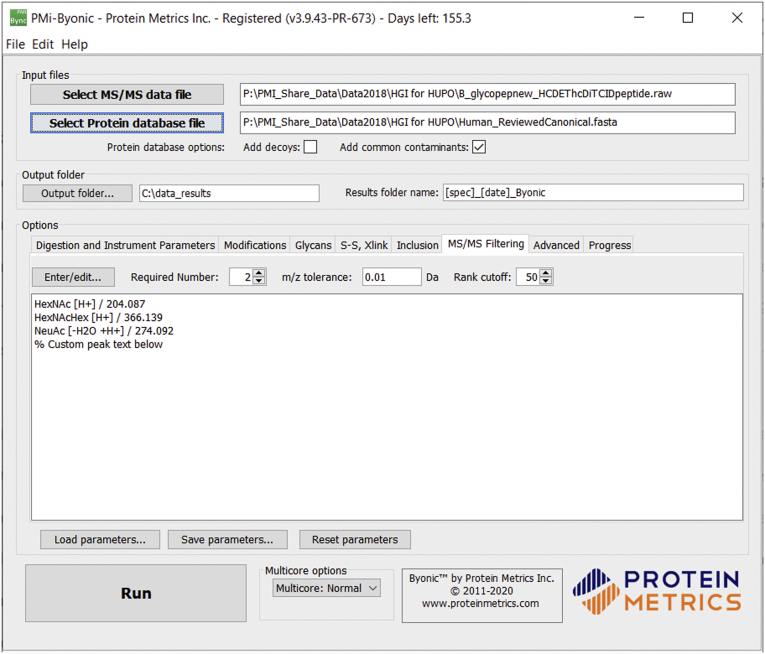
**Screenshot of Byonic user interface.** Byonic now includes an “Inclusion” tab for limiting the search to specific time and m/z ranges and an “MS/MS Filtering” tab (shown open) for limiting the search to spectra that contain specific *m/z* peaks. Here, the user specifies that a spectrum must contain at least two of 204.087, 366.139, and 274.092 ± 0.01 among its top 50 peaks.

As shown in Figure 1, the MS2 Peak Filtering controls include a pull-down menu with suggested filtering peaks, along with a box in which users can define their own. The syntax is simply a text label followed by a slash “/” and an *m/z* value. The user can also specify an *m/z* matching tolerance—the default value is 0.02 Thomsons—and a “required number” of peaks to match. For example, the user can require any one of three filtering peaks, or any two of four, and so forth. By default, MS2 Peak Filtering ignores peaks smaller than the 50th most intense peak in the spectrum, *i.e.*, the 51st peak does not count as a match; this “Rank cutoff” value is changeable on the “MS/MS Filtering” tab.

MS2 Peak Filtering can be used to find glycopeptides by filtering for common oxonium ion peaks such as 186.076 and 204.087. If run with a “normal” search, *i.e.*, a search with a tight precursor mass tolerance and automatic filtering to low FDR, oxonium ion filters will provide a speed-up. If run with a loose precursor mass tolerance and no FDR control (“Manual score cut” of −1000 and “Protein FDR” set to “No cuts” on the Advanced tab), filtered search can be used to find likely glycopeptide spectra (with preliminary, and often incorrect, assignments) that can be targeted for identification in subsequent searches. Less obviously, MS2 Peak Filtering can also be used to find all glycopeptides with the same peptide part by filtering for peaks for predicted or observed Y-ions such as Y0 (bare peptide) and Y1 (bare peptide plus core HexNAc) or predicted or observed peptide fragments such as a2, b2, or y-ions at likely cleavage points.

Inclusion List and MS2 Peak Filtering apply a binary filter, include/exclude, to MS2 spectra before they are searched against the protein database; these two new features do not affect Byonic scoring or annotation of included spectra. The third new feature, Custom Peaks, not yet released at the time of writing this article, gives the user a way to change scoring and annotation. The Custom Peaks feature uses a new command added to Byonic’s “fine control format”. For example, this modification rule

Woo/+346.1458@S,T | common2 | CustomPeaks{IsoTaG:347.1531, IsoTaG-36:311.1320, IsoTaG-90:255.0956}

directs the software to look for peaks at 347.1531, 311.1320, 255.0956 for candidate “peptiforms” (specific modification states of peptides) containing a “Woo” modification on S or T. If the spectrum does contain the peaks within the fragment mass tolerance, the score will increase; if the peaks are not found, the score will decrease. Byonic’s scoring uses both observed and predicted peak intensities in its scoring, and for reliable peaks such as a y-ion on the N-terminal side of proline or an oxonium ion at 274.092 for NeuAc with water loss, Byonic predicts high intensity. Byonic uses a medium predicted peak intensity for new peaks added *via* the “CustomPeaks” command. If the top-scoring peptiform does indeed contain a “Woo” modification, then the Byonic Viewer will annotate peaks within the annotation *m/z* tolerance of 347.1531, 311.1320, and 255.0956 as “IsoTaG”, “IsoTaG-36”, and “IsoTaG-90”. Byonic scoring and Byonic Viewer annotation are two different software modules; one obvious difference is that annotation can be adjusted (for intensity, *m/z* tolerance, and isotope peak spacing) after the Byonic search is complete.

Introduced in Byonic v3.9.4 (June, 2020) is the fourth new feature, Glycan Wildcard Search. Byonic has always included “wildcard” modification search. Wildcard search is a version of “error-tolerant”, “blind”, or “open” search—names and details vary—that allows a modification of any mass within a user-specified mass range. When the search scores a potential PSM, the mass of the wildcard is set by the difference between the spectrum precursor mass and the mass of the (possibly modified) peptide without the wildcard. Byonic’s UI for wildcard search on the Modifications tab includes a box labeled “Restrict to residues”. Capital letters input to this box refer to the 20 standard amino acids, and “n” and “c” specify peptide N- and C-terminus. The innovation is that “g” in this box now specifies a wildcard “on top” of a glycan. For example, the glycopeptide EEQYN∗STYR, with N∗ corresponding to asparagine with a glycan of composition HexNAc(4)Hex(4) (sometimes denoted G1) has neutral mass 2649.034 Da, so a wildcard of mass 22.001 would be added to the glycan before scoring this candidate peptiform against an MS2 spectrum with *z* = 3+ precursor with monoisotopic *m/z* 891.352 (corresponding to a neutral mass of 2671.035). If this candidate peptiform outscores all other candidate peptiforms, then Byonic will report a match of EEQYN[+1460.529]{+22.001}STYR, where 1460.529 specifies HexNAc(4)Hex(4). (Notice the curly braces for the wildcard mass delta.) The user might then decide that 22.001 is most likely sodiation, which has a theoretical mass of 21.982.

Glycan wildcard search differs from standard wildcard search because Byonic predicts oxonium ions and glycan loss ions such as Y0 (bare peptide), Y1 (bare peptide + HexNAc), etc. from collisional dissociation for a peptide with a glycan and glycan wildcard but not for a peptide with a nonglycan wildcard. Thus, a spectrum must have unusually good peptide backbone fragmentation to match EEQYN{+1482.530}STYR, a nonglycan wildcard, but an MS2 spectrum from collisional dissociation may match EEQYN[+1460.529]{+22.001}STYR based solely on oxonium and glycan loss ions.

### Software Tests

We used the rat brain peptidomics data from Secher *et al.* to test the speed gain from MS2 Peak Filtering for a large glycoproteomics search and to demonstrate that ordinary proteomics samples may contain unsuspected glycopeptides. We used the Uniprot proteome UP000002494 (*Rattus norvegicus*). We set Byonic for a nonspecific search, 6 ppm precursor tolerance, 20 ppm fragment tolerance, “Total common max” and “Total rare max” set to 1, and variable modifications

Oxidation/+15.994915 @ M | common1

Amidated/-0.984016 @ CTerm | common1

Gln->pyro-Glu/-17.026549 @ NTerm Q | rare1

Glu->pyro-Glu/-18.010565 @ NTerm E | rare1

Acetyl/+42.010565 @ NTerm | rare1

Deamidated/+0.984016 @ N | common1

Phospho/+79.966331 @ S, T | common1

We used an N-glycan database containing 100 N-glycan compositions that we had compiled based on mouse brain data from Trinidad *et al.* (17), and we used the O-glycan database called “O-glycan 9 common” that comes with the Byonic installation. Both N- and O-glycans were set to common1. We also checked the box for “Create focused database” to make a smaller FASTA file for subsequent searches to check glycosylated neuropeptide findings. We searched all the Copenhagen data, but for illustration purposes, we only use spectra from the file 20100805_Velos3_AnSe_Batch12faste_hypot_S3.raw. (Other fractions contain similar spectra.) The Wisconsin data from Ye *et al.* was used to confirm the glycopeptide findings from the Copenhagen data.

We used data set 3, the human T cell data with IsoTaG labels, to test the Custom Peaks feature. Byonic already has built-in support for IsoTaG, put in at the request of Christina Woo, so this data set enabled us to benchmark user-defined *versus* built-in custom peaks. The user-defined modifications were called Woo2 and Woo0 for heavy and light IsoTaG:

Woo2/+346.1458 @S,T | common2 | CustomPeaks{IsoTaG:347.1531, IsoTaG-36: 311.1320, IsoTaG-90: 257.11017}

Woo0/+344.1312 @S,T | common2 | CustomPeaks{IsoTaG:345.13855, IsoTaG-36: 309.1175, IsoTaG-90: 255.09562}

Equivalent rules using built-in keywords do not need mass numbers:

HexNAz2Si @ S,T | common2

HexNAz0Si @ S,T | common2

The two search strategies are close, but not identical, because the built-in modifications score only the peak at 347.1531 and use a large rather than medium intensity prediction. Moreover, Byonic makes the assumption that in positive-mode MS with QTOF/HCD fragmentation, any PTM with mass greater than 205 Da on S/T is labile and chargeable, so even before we added the Custom Peaks feature, Byonic would predict a peak at 347.1531. For this reason, we also used data set 4, human epithelial cell data with similar click chemistry but targeting very different biological modifications, prenylation rather than O-GlcNAcylation. We used the following modification rules on the concatenated .mgf file, which includes data from six .raw files, with all combinations of two different probes (YnF and YnGG) and three different capture reagents (AzRB, AzRTB, and Az3MRB). We ran Byonic with and without “CustomPeaks” for the most prominent fragment ions to test the advantage of custom peak prediction. AzRB and AzRTB give the same mass deltas so the following rules suffice:

AzRB-YnF/+459.2974 @ C | common2 | CustomPeaks{AzRB-YnF:460.3047,AzRB-YnF+H2S:494.2913}

AzRB-YnGG/+527.3600 @ C | common2 | CustomPeaks{AzRB-YnGG:528.3673}

Az3MRB + YnF/+629.4393 @ C | common2 | CustomPeaks{Az3MRB-YnF:630.4466}

Az3MRB + YnF/+697.5019 @ C | common2 | CustomPeaks{Az3MRB-YnGG:698.5092}

There are also low-mass peaks from geranylgeranylation fragments (31), *e.g.*, 120.080 and 129.103, but we did not include these as custom peaks, because they are at the low end of the *m/z* range and have low intensity in the spectra from data set 4.

We used CD16a/FcγRIIIa protein data to test if Glycan Wildcard Search could discover glycans even in a one-protein data set that had been thoroughly analyzed by experts. Finally, we ran Glycan Wildcard Search on the many-protein HUPO HGI plasma data to evaluate interpretability of spectrum assignments that include wildcard masses.

For wildcard searches, both glycan and “ordinary”, as well as “open search” (wide precursor mass tolerance (32)), we usually set “Precursor isotope off by x” on the Advanced tab to “No error check”, because a wildcard of 1 or 2 Da can compensate for an incorrectly called precursor monoisotopic mass. For the computational experiments reported here, we used “Automatic score cut” on the Advanced tab, because our purpose is to illustrate software features, rather than to conduct a thorough analysis of any data set. For thorough analysis of a simple sample, one might choose “Show all N-glycopeptides” and “Manual score cut” with a low score cutoff such as -1000, and one might even leave “Add decoys” unchecked, to find more PSMs for manual evaluation.

## Results

### Search Speedup and Sensitivity

The search described above on the Copenhagen data took 23 h and 30 min using six cores of an 8-core laptop (Intel Xeon CPU running at 2.8 GHz) on a single file (20100805_Velos3_AnSe_Batch12faste_hypot_S3.raw) with 30,882 scans (both MS1 and MS2). The time improved to 9 h and 16 min, about 2.5× speedup, with MS2 Peak Filtering requiring any three of the following five peaks: 138.055, 186.076, 204.087, 274.092, and 366.139, which are C_7_H_8_NO_2_ (a fragmentation product of HexNAc), HexNAc minus water, HexNAc, NeuAc minus water, and HexNAc(1)Hex(1), respectively. Every one of the 22 glycopeptide spectrum assignments with posterior error probability (PEP 2D) at most 0.001 found by the unfiltered search was also found by the filtered search, meaning that no confidently assigned glycopeptide spectrum was lost because of filtering. The reverse is automatically true: any peptide-spectrum match (PSM) made by the filtered search will also be made by the larger, unfiltered search and will give the same Byonic score. PEPs, however, may change, because the filtered search has fewer “true” and “false” PSMs for postsearch machine learning (33, 34). Indeed, in this case, the filtered search gave 25 glycopeptide matches with PEP at most 0.001.

The 2.5× speedup is surprisingly small. Only 229 out of 20,943 spectra pass the Peak Filtering test, so one might expect a speedup around 90×. The actual speedup is much less because of reduced parallelism for small sets of spectra. Byonic breaks a search into “chunks”, each containing 500 to 2000 spectra, so the filtered search used only a single CPU core. Reducing the minimum size of a chunk from 500 to 50 did not improve the speed, because with such a small number of spectra, most of the computing time is spent generating billions of candidate glycopeptides rather than scoring the relatively small number of spectra. On other searches, the speedup from MS2 Peak Filtering varied from 1× (meaning no speedup at all) to more than 20×.

### Unsuspected Glycopeptides

**Fig. 2:**
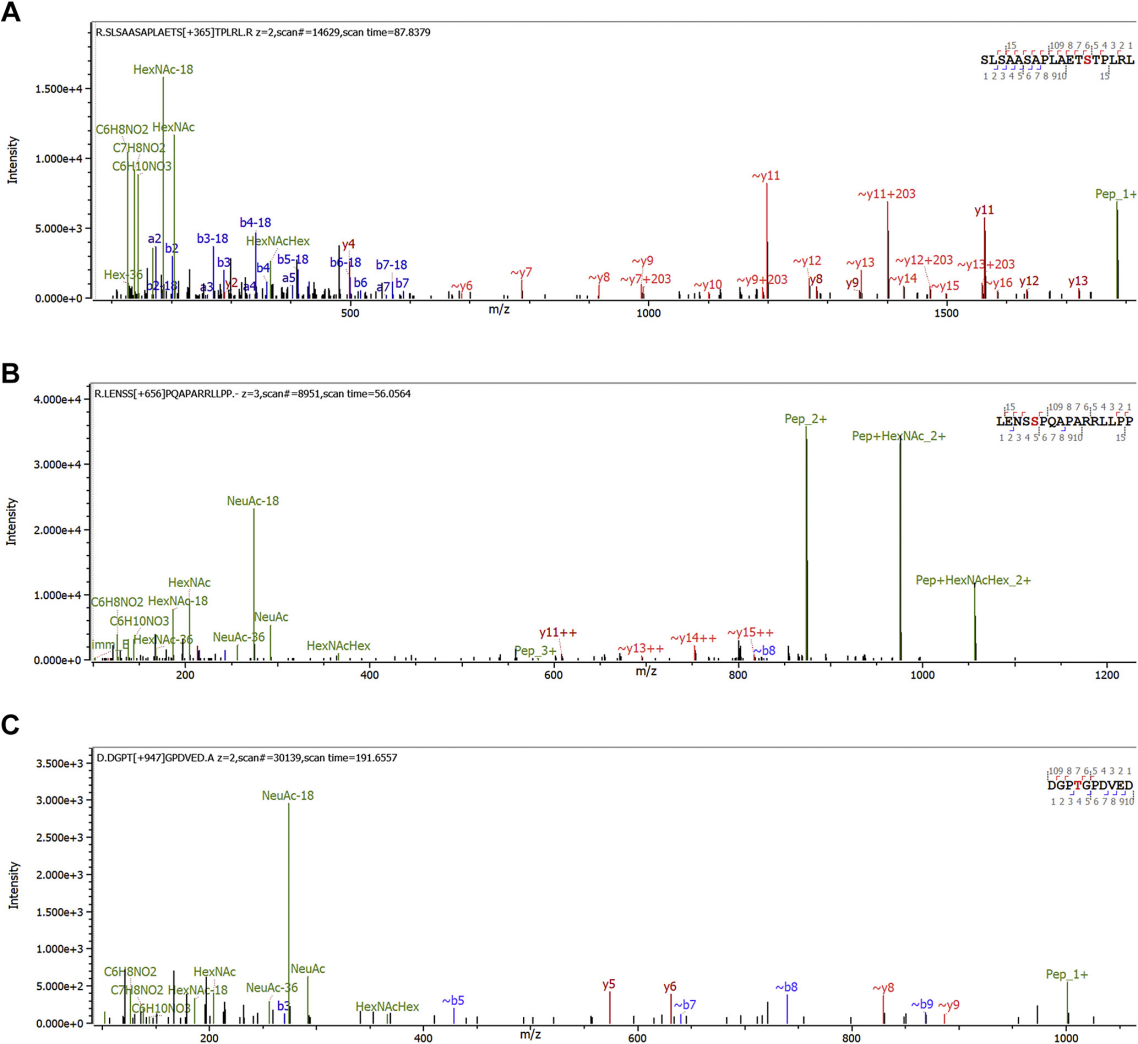
**Glycosylated neuropeptides from proSAAS in Copenhagen data discovered using MS2 filtering.***A*, little SAAS with O-linked glycan localized to TST by ∼y + 203 peaks, that is, y-ions with base GalNAc, (*B*) Big LEN, and (*C*) an unnamed peptide, residues 171 to 180. Fragments are matched to ±20 ppm.

Figure 2 and supplemental Figs. S1 and S2 show glycosylated neuropeptides from the protein proSAAS from this search. “Little SAAS” and “Big LEN” in Figure 2, *A*–*B* are known neuroptides (27), but the peptide in Figure 2*C* does not seem to appear to have a name. The glycosylated peptides are all of low abundance relative to the unmodified peptides, except for the peptide in 2(c) which was not observed unmodified, probably because of the data acquisition method, which avoided acquiring MS2 scans of singly charged peptides. See the table in supplemental Fig. S2. To our knowledge, glycosylation has not been reported on these peptides before. Supplemental Figs. S4–S11 show other possible glycosylated neuropeptides found in the Copenhagen data.

**Fig. 3:**
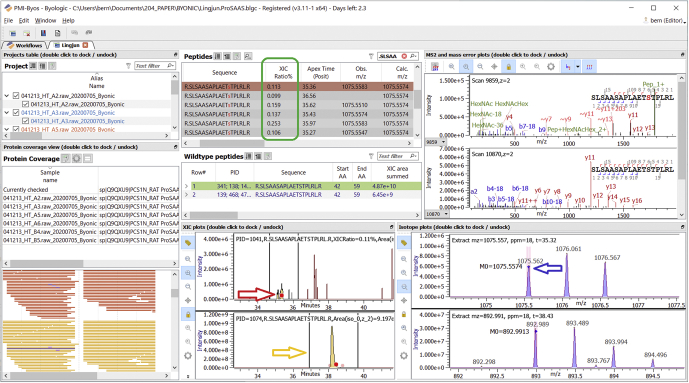
**Little SAAS glycopeptides in Wisconsin data.** Here, we show the same glycopeptide as in Figure 2*A* in Wisconsin data from hypothalamus of fed (B files) and unfed (A files) rats. As shown in the “XIC Ratio%” column (*green box*), the glycosylated peptide has only about 0.1 to 0.25% of the abundance of the unmodified “wildtype” peptide, considering only the *z* = 2+ form of each peptiform. We transferred the identification between runs inside Byos software, because not all runs included an identified MS2 scan. Byos allows easy comparison of extracted ion chromatograms (XICs), MS1, and MS2 plots. Dots in XICs (*red arrow*) show the times of the displayed MS2’s. Time limits for XIC quantification (*gold arrow*) are adjustable. Dots in MS1 plots (*blue arrow*) show calculated monoisotopic *m/z*.

The Wisconsin data set confirmed all the proSAAS glycosylation sites from Figure 2, except for *2*(c), which was not observed in the Wisconsin data. A much longer, unmodified peptide with sequence DGPTGPDVEDAADETPDVDPELLRYLLGRILTGSSEPEAAPAPRRL was observed, which tends to confirm the interpretation that shorter unmodified peptides such as DGPTGPDVED were not observed in the Copenhagen data because of the data acquisition method. Figure 3 shows the same Little SAAS glycopeptide as in Figure 2*A* but identified from the hypothalamus samples in the Wisconsin data set and compared across six rats. Supplemental Fig. S3 shows the same Big LEN glycopeptide as in Figure 2*B*, compared across rats. With so few experimental animals (4 unfed and 2 fed rats), it is impossible to say whether there is any difference in glycosylation between the unfed and fed conditions.

### Custom Peak Scoring and Annotation

**Fig. 4:**
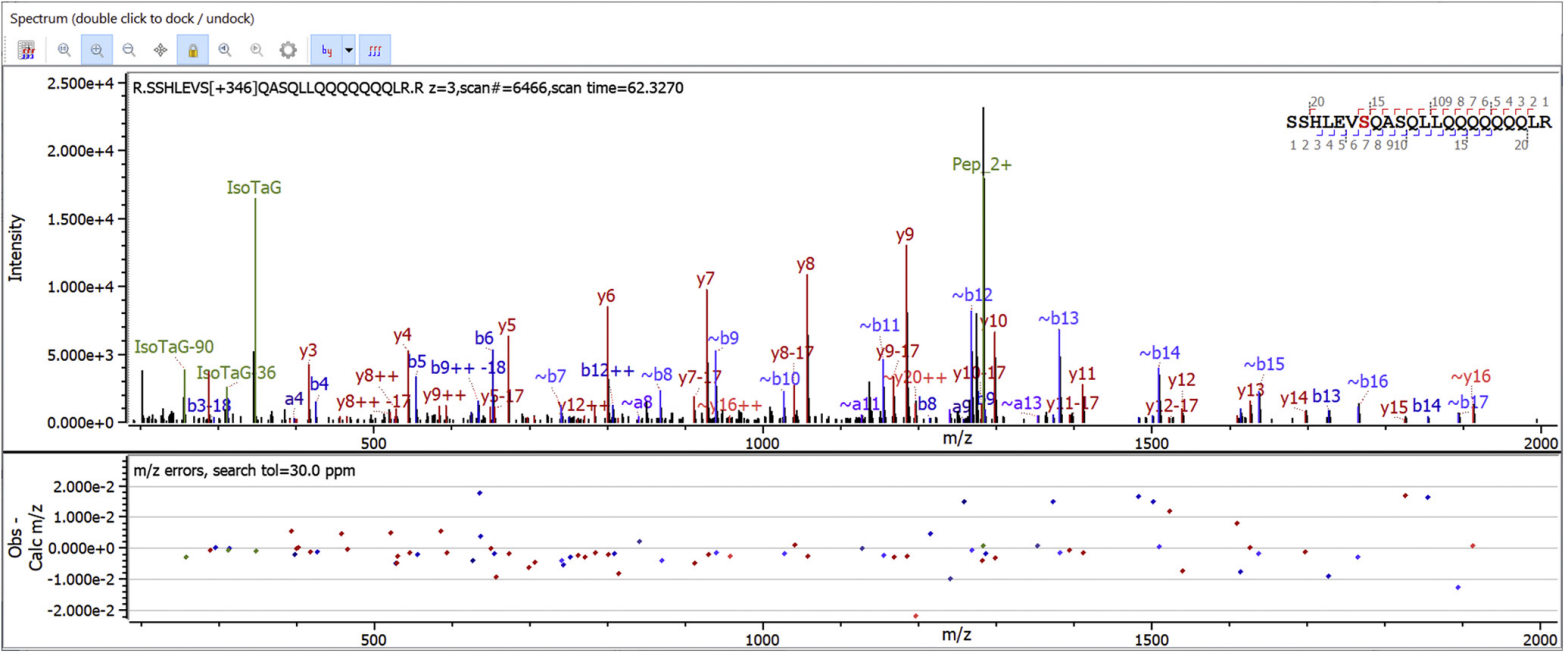
**User-customized scoring and peak labels.** The next version of Byonic (September, 2020) will include a way for software users to specify new PTMs with peaks to be scored and annotated. The spectrum above shows a peptide from NCOR1_HUMAN (Nuclear receptor corepressor 1) with an isotopically labeled version of O-GlcNAc called IsoTaG with atomic formula C_13_H_18_D_2_N_4_O_7_ and mass 346.146. The scored and annotated peaks at 257.110, 311.132, and 347.153 are specified by the user. Byonic makes the assumption that large-enough PTMs on S/T are labile, and it labels fragment ions missing their labile modifications with ∼ as in ∼b8 to ∼b16 above. PTMs, posttranslational modifications.

Figure 4 demonstrates the Custom Peaks feature. User-defined scoring for “Woo2” and “Woo0” (unknown to Byonic) and built-in scoring using HexNAz2Si and HexNAz0Si (predefined keywords for the same PTMs) gave essentially identical results on 341_iso_glycan_trypsin_scout.raw: 489 PSMs containing “Woo” and 1622 total PSMs, and 482 PSMs containing “HexNAz” and 1616 total PSMs, with “PEP 2D” at most 0.01, that is error probability 1% or less. (This .raw file also includes product-dependent electron transfer dissociation (ETD) scans, which are important for O-linked glycosylation site localization; interestingly, most of the ETD scans include small peaks at 347.15 as well.) As described above, both searches score the peak at 347.1531, and additional, highly correlated, peaks like 257.11017 add only a little sensitivity, so it is not surprising that the search results are almost identical.

On the epithelial cell data set, the search with Custom Peaks gave 37,391 PSMs and 8847 unique peptides, and an otherwise identical search without Custom Peaks gave 37,307 PSMs and 8820 unique peptides, with “false discovery rate (FDR) 2D” at most 0.01, that is, with estimated percentage of false positives 1%. (This is a less strict requirement than 1% error probability for each PSM individually.) These results again appear to be essentially identical, but this time, there is an important difference. The search without Custom Peaks gave 120 PSMs and 41 unique peptides with prenylation, which is a rare but important group of posttranslational modifications, and the search with Custom Peaks gave 148 PSMs and 56 unique peptides with prenylation, an improvement of 37% at the peptide level. Here, we counted the same prenylated peptide captured with AzRB/AzRTB and Az3MRB as unique peptides because the “clicked” modifications are distinct. Expert curation revealed that the FDR on prenylated peptides, which tend to be short peptides with poor fragmentation, is clearly higher than 1%, but we still consider Custom Peaks a success because the search yielded more candidates for curation and the peak annotations made curation faster and more accurate. The peaks for AzRB/AzRTB or Az3MRB clicked to YnGG or YnFF are generally intense peaks, so manual curation discarded all PSMs without the expected peak. Supplemental Figs. S12–S15 show some prenylated peptides not reported in the original publication (29).

### Unanticipated Glycans From Glycan Wildcard Search

**Fig. 5:**
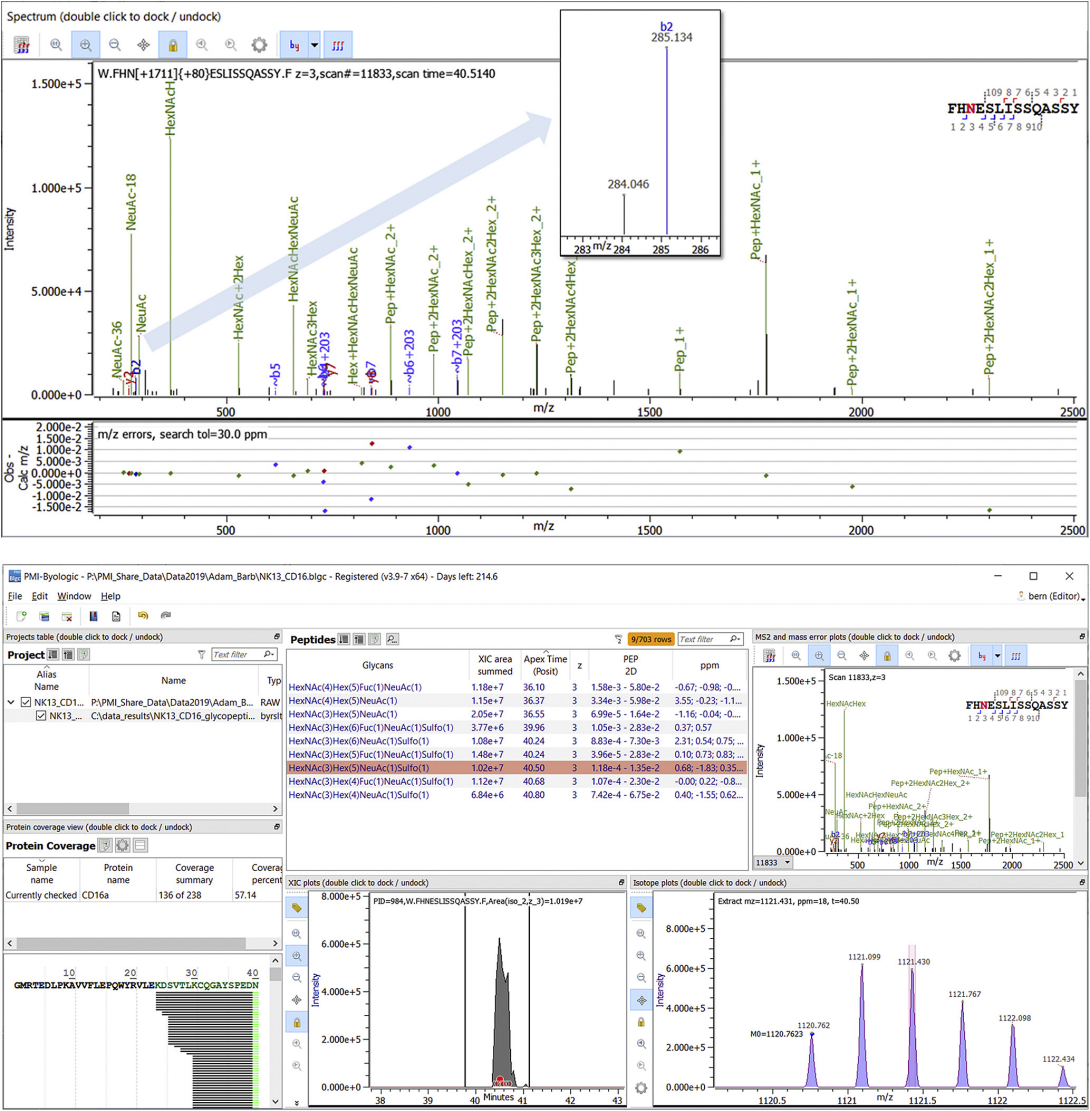
**Sulfated glycans on NK CD16a/FcγRIIIa.** The spectrum (*top*) shows N-45 with HexNAc(3)Hex(5)NeuAc(1) and a wildcard with mass 79.9576, close to both sulfation, 79.9568, and phosphorylation, 79.9663, which give mass errors of 0.2 and 2.7 ppm for this 3360-Da glycopeptide. The lower ppm error, consistent with other high-scoring matches in this data set, and the small peak at 284.046 (which the next release of the Byonic Viewer will annotate for glycans containing both “HexNAc” and “Sulfo”) provide evidence that the modification is sulfation. After discovery *via* wildcard search, sulfated glycans can be added to the glycan database for a more sensitive known-glycan search and quantitation by Byos (*bottom*). The glycan compositions indicate mostly hybrid structures with a single GlcNAc-Gal-NeuAc antenna, often sulfated. Glycopeptides with sequence FHN[+Glycan]ESLISSQASSY and nonsulfated glycans have total area under curve (AUC) of 4.38 × 10^7^. The same peptide with sulfated glycans has total AUC of 5.76 × 10^7^.

Figure 5 demonstrates the use of the new glycan wildcard modification. The glycopeptide shown in Figure 5 was first discovered by a Byonic “hack” that searched for glycopeptides with N-linked glycans by using a generic glycan database along with a wildcard modification on S/T. Glycopeptides containing glycans not in the glycan database will often match the correct peptide with an incorrect glycan in the database, along with a wildcard correcting the total mass to the precursor mass of the spectrum. The new glycan wildcard feature speeds up the hack and elevates it to an officially supported Byonic search mode. One might guess that the speedup should be about 20× for a large search because only about one in 20 S/T residues is preceded by N in the −2 position. Experimentally, we obtained a speedup of 2.5× (from 75 to 31 min) on B_glycopepnew_HCDEThcDiTCIDpeptide.raw, one of the spectrum files from the HUPO HGI plasma glycoproteomics study, using a glycan database with 132 N-linked glycans and a glycan wildcard with mass in the range −50 to 300 Da. The reason that the speedup is not 20× is that in a glycopeptide search, peptides with Nx{S/T} motifs constitute much of the search, and these peptides produce roughly the same number of peptiforms with a glycan wildcard as with an “ordinary” wildcard on S/T. Also note that a wide wildcard range will include near-duplicate peptiforms, *e.g.*, the same peptide with HexNAc(2)Hex(4) and with HexNAc(2)Hex(3) + 162.13 wildcard. To reduce duplication, the wildcard range should be narrowed to roughly the size of the gaps between the masses of glycans in the glycan database. For completeness, we mention one more option related to Glycan Wildcard Search: an open search allowing a wide precursor mass tolerance, *e.g.*, ±250 Da. An open search is at least as fast as a glycan wildcard search with similar mass range on the peptides with Nx{S/T} motif, but the results may require more time and effort to interpret, as we discuss below.

Glycan Wildcard Search does not give a speedup for O-linked glycan search. With a glycan wildcard, each S/T will be considered with a glycan or a glycan and a wildcard, and with an ordinary wildcard restricted to S/T, each S/T will be considered with a glycan or a wildcard but not both; the ordinary wildcard search is actually smaller, and an open search is probably still smaller (depending upon the number of S/T’s in the peptides). Also note that, as with Byonic’s original wildcard modification, there is a limit of one glycan wildcard per candidate peptiform.

Glycan Wildcard Search (or open search) pairs well with MS2 Peak Filtering to find all the MS2 spectra of glycopeptides with the same peptide part. As a demonstration, we searched for all glycopeptides with peptide YLGNATAIFFLPDEGK from alpha-1-antitrypsin, by filtering for spectra containing three of six peaks: 204.087 (HexNAc oxonium ion), 545.257 (y5), 658.341 (y6), 942.478 (y8), 1755.896 (Y0), and 1958.974 (Y1). We allowed a wildcard of mass from −50 to 700 Da. The search on the HCD spectra of B_glycopepnew_HCDEThcDiTCIDpeptide.raw using a “focused” protein database containing 246 target and decoy proteins took less than 1 min, and every returned PSM had peptide part YLGNATAIFFLPDEGK. (Notice that ETD spectra would require different filtering peaks). Supplemental Fig. S16 shows one high-scoring and interpretable PSM.

We note that in data from glyco-enriched samples such as B_glycopepnew_HCDEThcDiTCIDpeptide.raw, many MS2 spectra, even those of ordinary peptides, contain small peaks at 204.087, 274.092, and 366.139 from background glycopeptides. A “Rank cutoff” of 20 or 30 on filtering peaks should eliminate most ordinary peptide spectra with acceptably low loss of glycopeptides.

Finally, we report on the interpretability of N-linked glycan wildcard PSMs. Glycan wildcard search, like open search, generally requires expert interpretation to map PSMs with wildcards or large precursor mass errors to likely explanations such as adducts, nonspecific termini, known but unanticipated PTMs, and—on rare occasion—previously unknown PTMs. Glycan wildcard search differs from open search in that only peptides containing the consensus sequence motif will be allowed large precursor mass errors, thus reducing the time for expert curation.

We examined the top-scoring PSMs from a search of B_glycopepnew_HCDEThcDiTCIDpeptide.raw using a glycan wildcard with mass from −30 to +205 Da and the 246-protein focused database. Out of 547 PSMs with error probability (PEP 2D) at most 0.01, the most common glycan wildcard masses, to the closest integer, were 201 to 205 Da (55 occurrences), 161 to 164 (47 occurrences), −1 to 1 (44 occurrences), 62 to 64 (37 occurrences), 37 to 39 (27 occurrences), and 21 to 22 (14 occurrences), which we interpret as HexNAc, Hex, precursor mass out of tolerance, copper(?), calcium or potassium, and sodium, respectively. Supplemental Fig. S17 gives a histogram of wildcard masses. Supplemental Fig. S18 gives evidence for copper as an explanation for the 62 to 64 Da mass deltas. For many PSMs with a glycan wildcard with mass close to 162 or 203, the glycan with the correct composition was indeed in the glycan database, but the match was made to a wildcard glycan rather than to the correct glycan because of large error in the precursor monoisotopic mass, which is often inferred from incomplete isotope clusters with low signal-to-noise ratio. There are, however, a number of PSMs that could be from modified glycans or glycans not in the database. See supplemental Fig. S19.

## Discussion and Conclusions

The work reported here suggests some subjects for future study in both glycobiology and glycoproteomics software. On the biological side, the work reported here found O-linked glycosylation on neuropeptides, similar to corresponding recent discoveries of O-linked glycosylation on human and mouse insulin (35) and O-linked glycosylation on crustacean neuropeptides (36). In all cases, the glycosylated peptides are of low abundance relative to the wildtype peptide, so the glycosylated forms may simply be a harmless product of some nonspecificity—“leakage”—of GalNAc transferases, which initiate most O-linked glycosylation in secreted proteins. It is known, however, that O-linked glycosylation can protect biologically active peptides from protease activity and can be manipulated for greater bioavailability (37, 38), so the small percentage of glycosylated neuropeptides may play a functional role by giving the molecules a range of lifetimes.

CD16a/FcγRIIIa glycosylation plays a key role in antibody-dependent cellular cytotoxicity. Glycosylation on N-162 enhances binding to antibodies lacking core fucosylation (39) and hence promotes antibody-dependent cellular cytotoxicity, but glycosylation on N-45 inhibits this binding (40). These findings have stimulated research into cell type–specific and site-specific glycosylation analysis of FcγRIIIa (30, 41), and it has recently been shown that an L/H polymorphism at residue 48 can influence N-45 glycosylation (42). Our observation of sulfation on N-45 may be of interest because sulfation changes the charge distribution and often the binding affinity of glycans (43).

On the software side, the work reported here continues our effort to support and improve glycopeptide search within a general-purpose proteomics search program. There are several advantages to building an integrated proteomics tool, rather than a number of special purpose tools, especially for a commercial product: single user interface (UI), code reuse, larger market size, and wider education and training. The finding of unsuspected glyconeuropeptides in unenriched samples from two different laboratories, along with a previous finding on MHC Class I bound glycopeptides (44), demonstrates the utility of glycopeptide search in a conventional proteomics search tool. Byonic itself is integrated into a larger package called Byos (Figs. 3 and 5), which provides quantitation, comparison, and reporting, all crucial for contract research organizations and biopharmaceutical companies. Contract research organizations and drug companies are not generally at the forefront of glycobiology research, unless the targeted health problem is itself a disease of glycosylation, but they do have to analyze glycosylation on drug molecules and targets, and this work is often performed by nonspecialists.

An obvious next task for software development is to improve the speedup provided by MS2 Peak Filtering. One possibility is to preprocess the spectra for the predefined filtering peaks such as 204.087, 274.092, etc., rather than to check peaks anew for each search. Another, similar, possibility is to cache the peak filtering results of the first search, which would allow user-defined as well as predefined peaks to be included in the speed-up. Glycan Wildcard Search would also benefit from speed improvement. This new feature is currently quite usable for searching for glycopeptides with only N-linked glycans, but it is slower than ordinary wildcard or open search for unanticipated O-linked glycans.

Planned improvements include specialized FDR control for glycopeptides and incorporation of retention time information as in (45). Currently, Byonic’s machine learning for FDR estimation and control considers two informational levels, PSMs and proteins (46). We experimented with a middle informational level in the initial research (34) by including statistical features that connect modification forms of the same peptide, but this level was not included in the current commercial product. (At that time, even two levels of FDR control was unusual, as most laboratories used either the “two peptide rule” or no filtering at all to control protein-level FDR.) This middle level is likely to be even more effective for glycopeptides than for phosphopeptides, especially if retention time is taken into account. Generally speaking, if the best PSM in a “block” of glycopeptides with the same peptide part has a strong Byonic score and the block has close-enough elution time in reverse phase, with sialic acids shifting elution time slightly later than neutral monosaccharides, then it is likely that the entire block is correct. If the best PSM in a block is weak, then it is likely that the entire block is incorrect. We are working on these improvements now, and will have them ready for a future Byonic release.

## Conflict of interest

Authors declare no competing interests.
