# Supplementary material for: Peak Filtering, Peak Annotation, and Wildcard Search for Glycoproteomics
Source: Mol Cell Proteomics. 2020 Dec 8;20:100011. doi: 10.1074/mcp.RA120.002260 (PMC8724605; doi:10.1074/mcp.RA120.002260)
Supplement: Supplemental Figures S1–S19 [file mmc1.pdf]

Peak Filtering, Peak Annotation, and Wildcard Search for Glycoproteomics

Supplemental Information

Marshall Bern*, Abhishek Roushan, Doron Kletter, K. Ilker Sen, Eric Carlson

Protein Metrics Inc., Cupertino, CA 95014

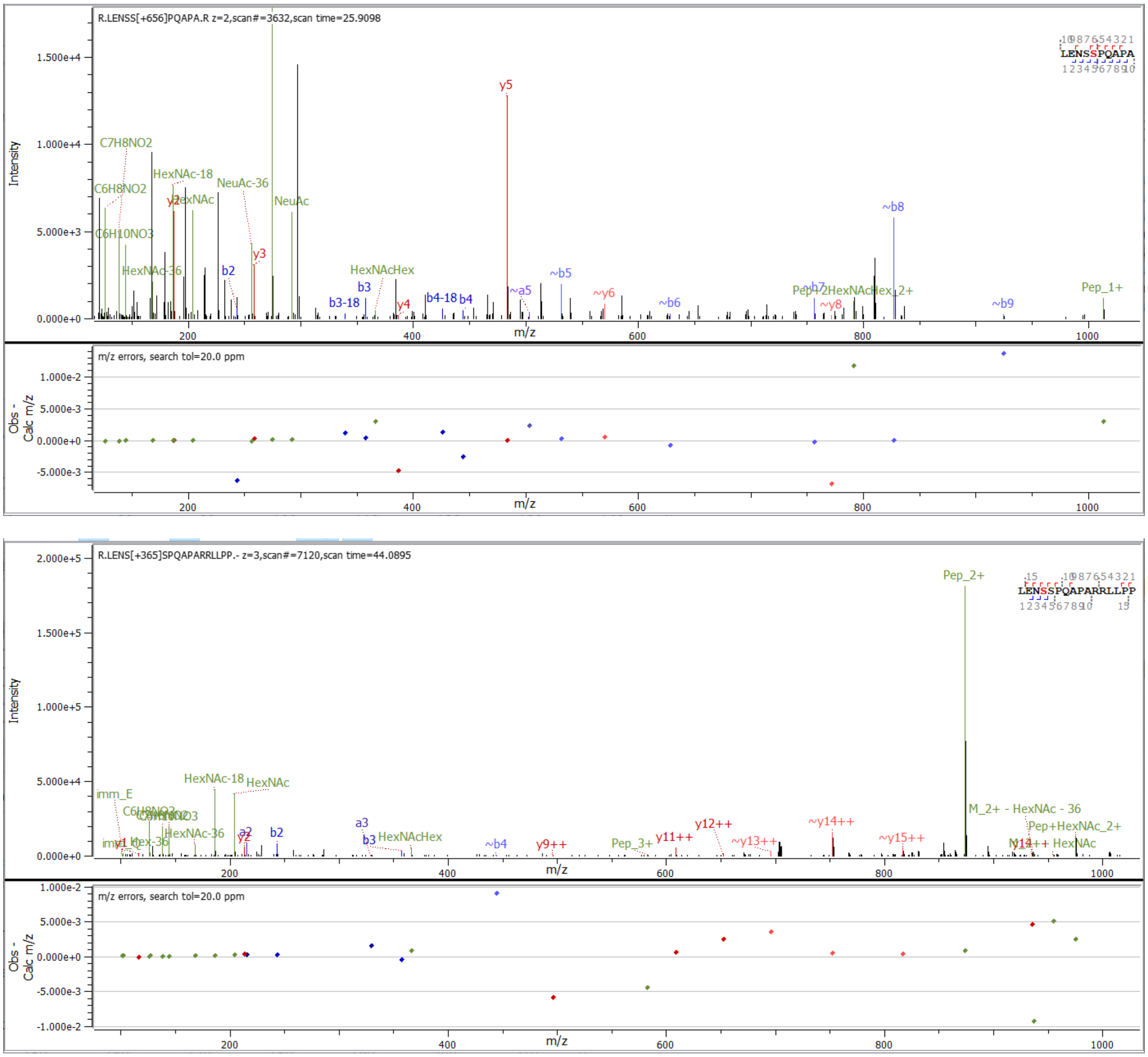

Figure S1. Little LEN and Big LEN glycopeptides from ProSAAS in the Copenhagen data set, ProteomeXchange PXD002431, 20100805_Velos3_AnSe_Batch12faste_hypot_S3.raw.

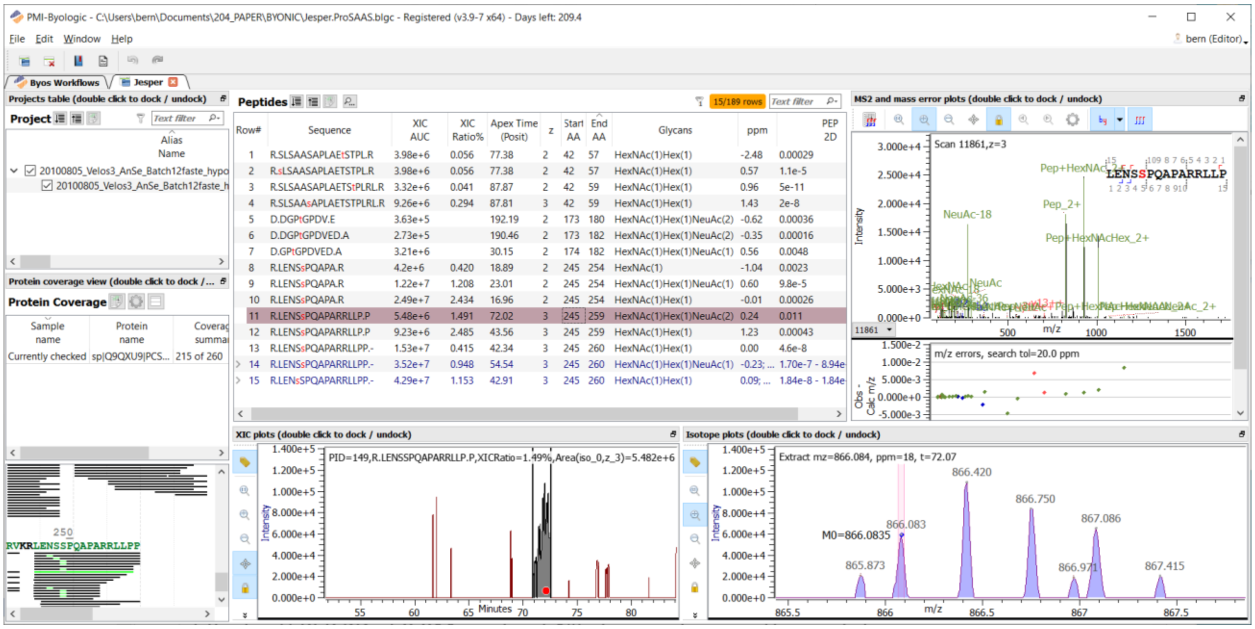

Figure S2. ProSAAS glycoforms from 20100805_Velos3_AnSe_Batch12faste_hypot_S3.raw. The Little LEN peptide selected above has the worst (highest) PEP 2D of 0.011 or about 1% chance of error. We show this peptide to give a sense of the threshold for acceptance of a spectrum assignment.

XIC Ratio % reports the XIC integral of the peptide in the given modification state to the sum of XIC integrals of all “peptiforms” with the same peptide sequence. All of the glycosylated species are low-abundance. Glycosylated forms account for about 4% of Little LEN, 2.5% of Big LEN, and 4% of “Medium LEN”, that is, LENSSPQAPARRLLP. The peptides containing the GPTG glycosylation site, rows 5 – 7 in the table, do not appear without glycosylation, probably because the data acquisition method did not select z=1+ precursors for MS2, so these peptides do not have an XIC Ratio %. Also notice the end-of-chromatography elution times for rows 5 and 6.

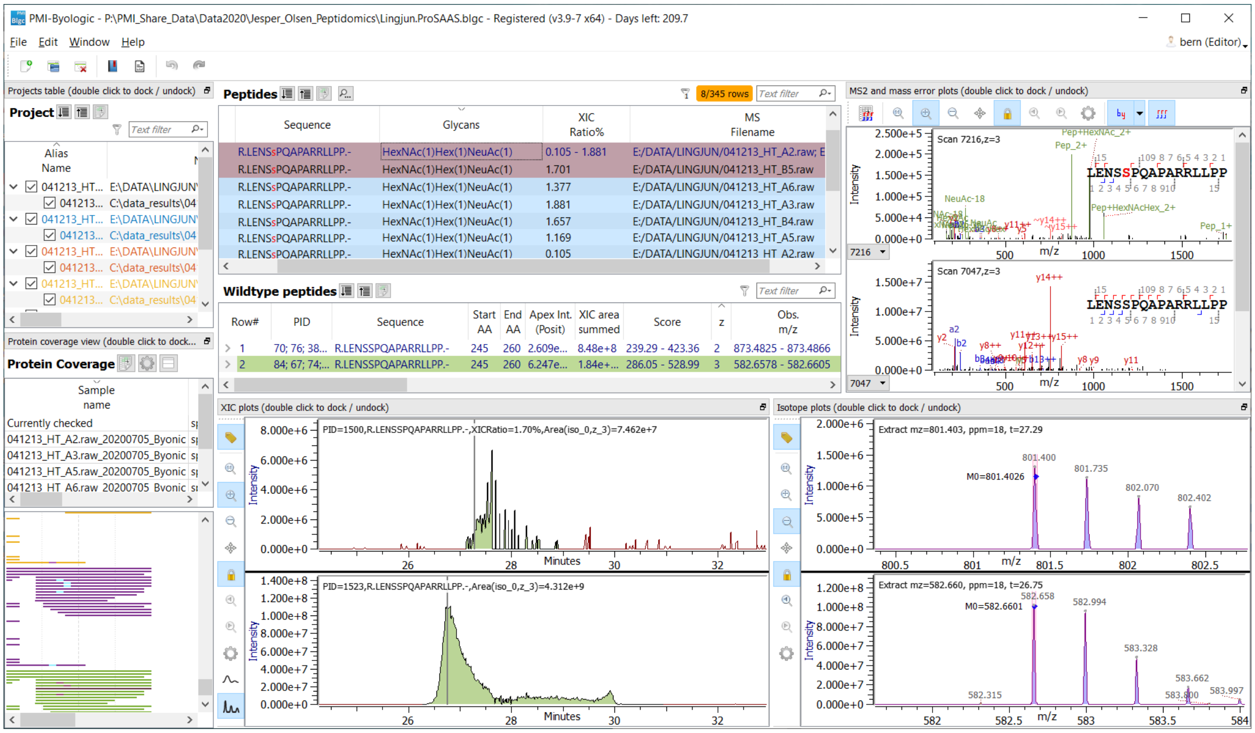

Figure S3. Big LEN glycopeptide compared across six Wisconsin data .raw files (all the files in ftp://massive.ucsd.edu/MSV000080106 with names starting “041213_HT”) from six different rat brains. The glycopeptide was identified by MS2 in only two of the data files but then transferred between runs using “Add Missing Via Existing Peptides” in Byos.

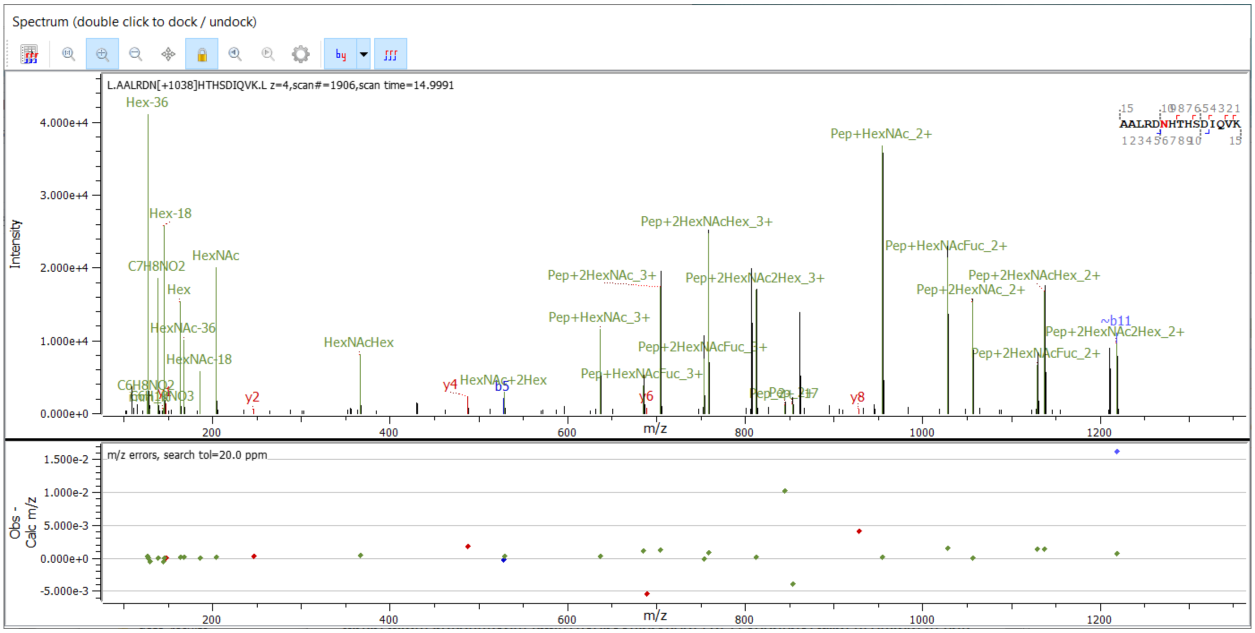

Figure S4. Phospholipase D3 peptide, positions 380 – 393. Figures S4 – S11 show a variety of glycopeptide PSMs from the Copenhagen data set with “PEP 2D” error probabilities below 1%.

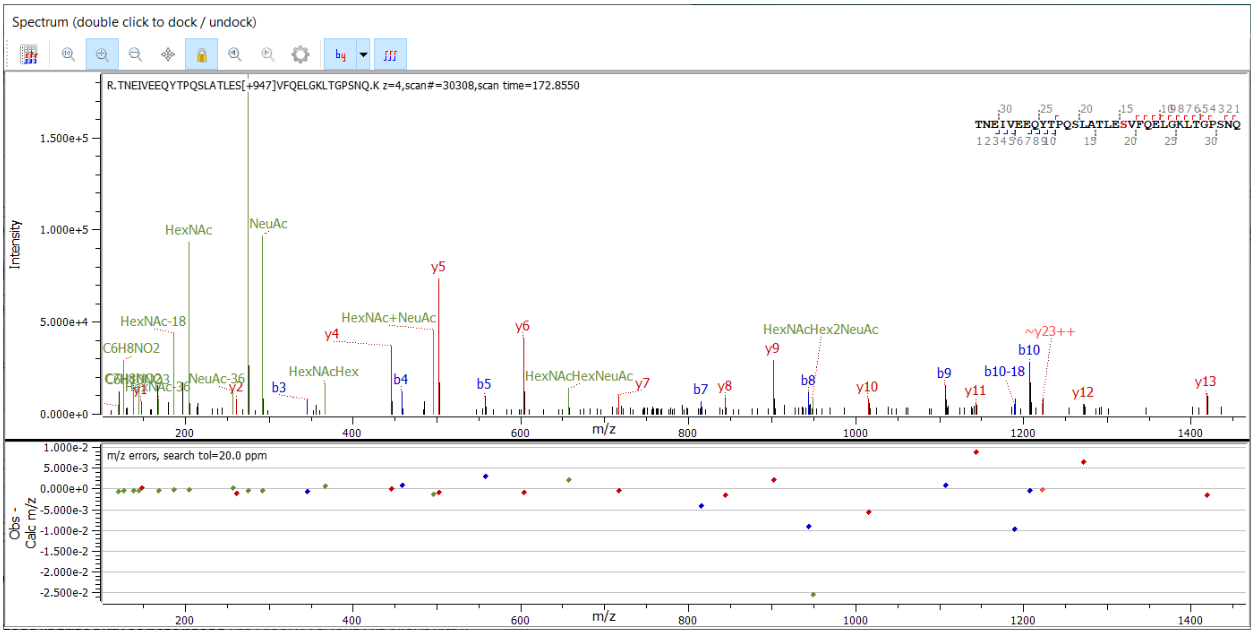

Figure S5. Secretogranin-2 isoform X1.

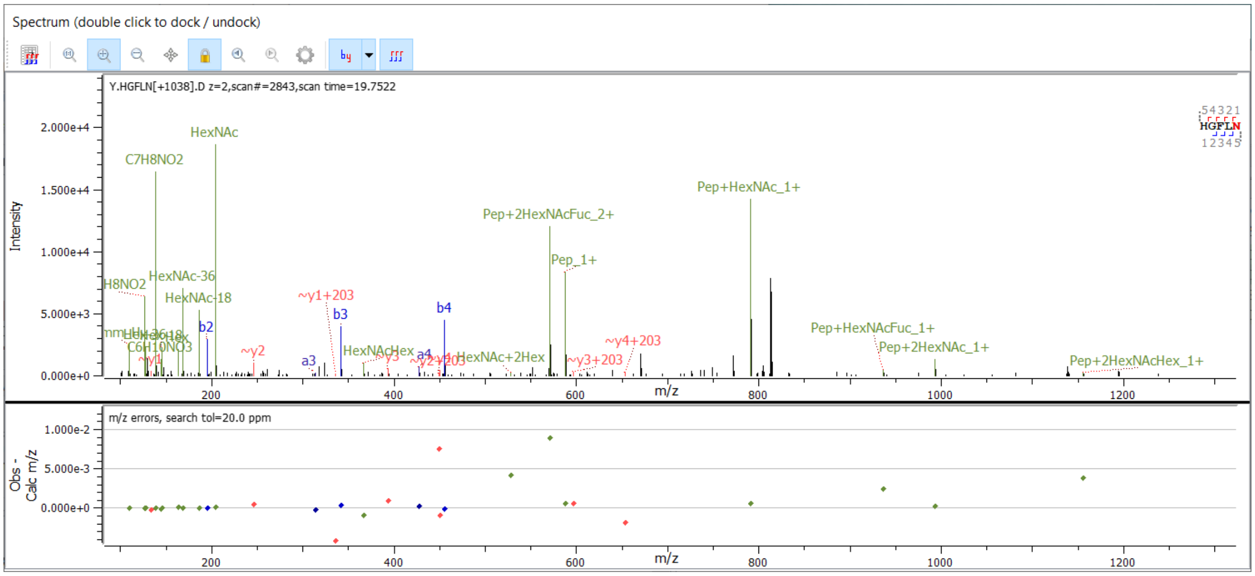

Figure S6. Peptide with an N-linked glycosylation (fucosylated N-glycan core). This short peptide appears in Lysosomal acid phosphatase isoforms with the C-terminal N in an N-glycosylation sequon, and in two other human proteins (furin and epoxide hydrolase 3) without the sequon. Short peptide matches such as those in S6, S7, and S9 must be considered somewhat ambiguous as to protein of origin.

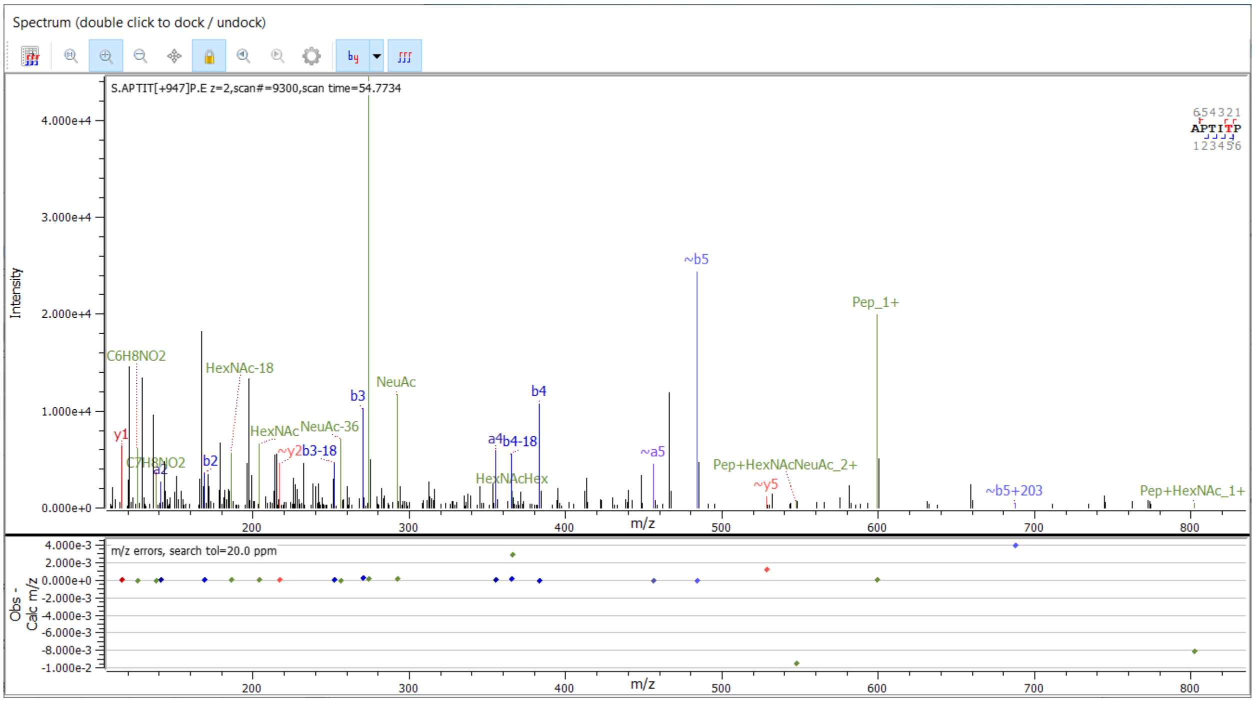

Figure S7. Secretogranin-1 peptide.

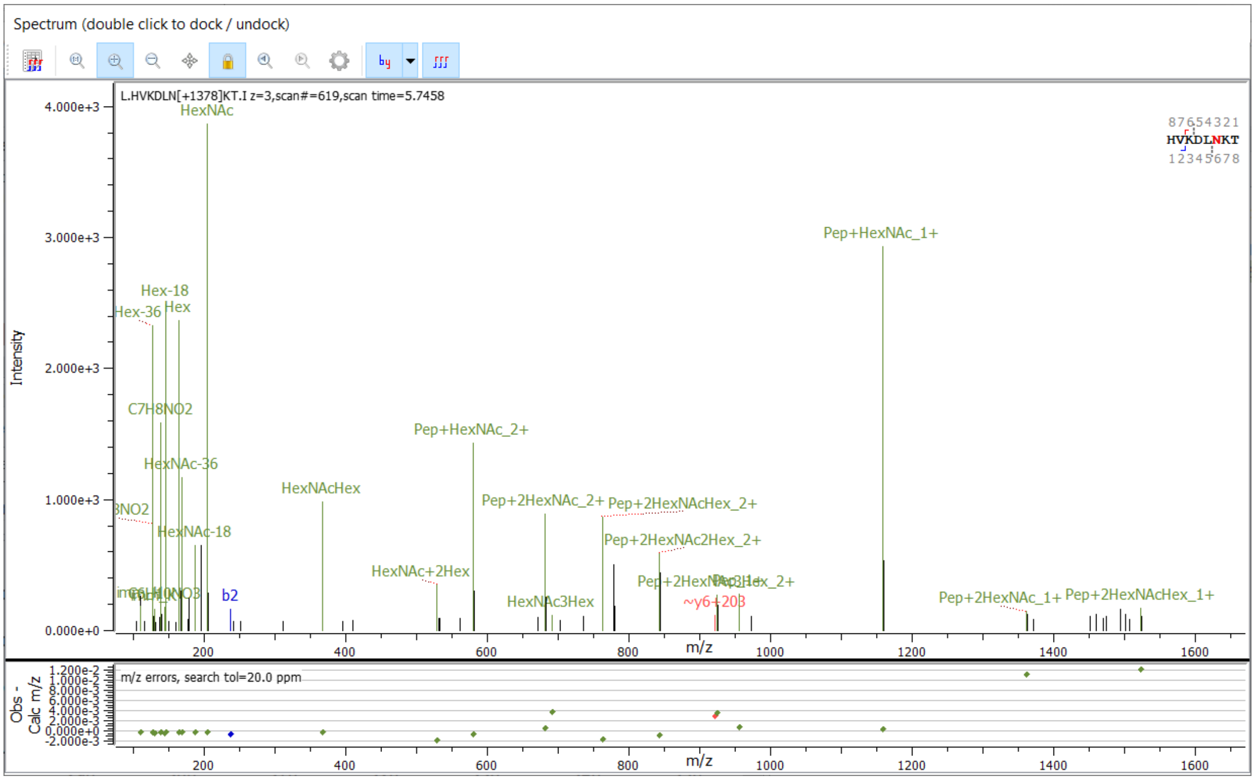

Figure S8. Legumain isoform X2.

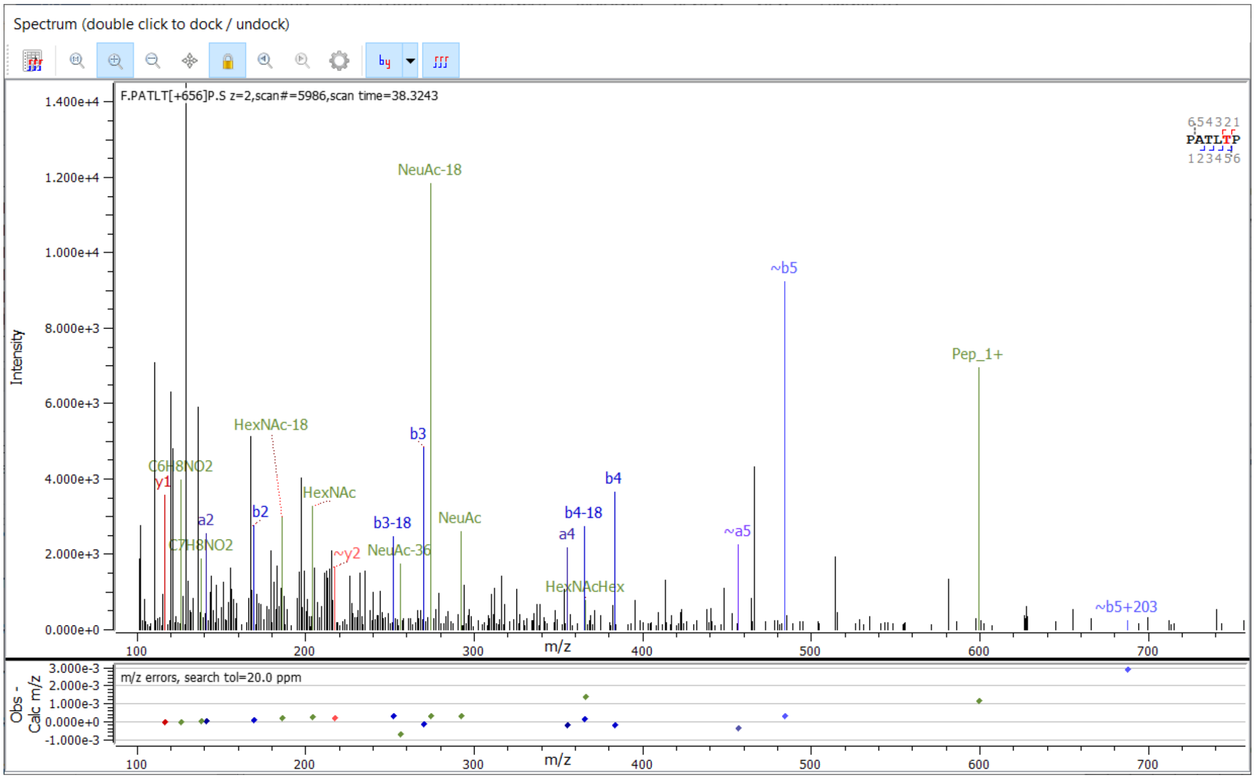

Figure S9. Aftiphilin peptide.

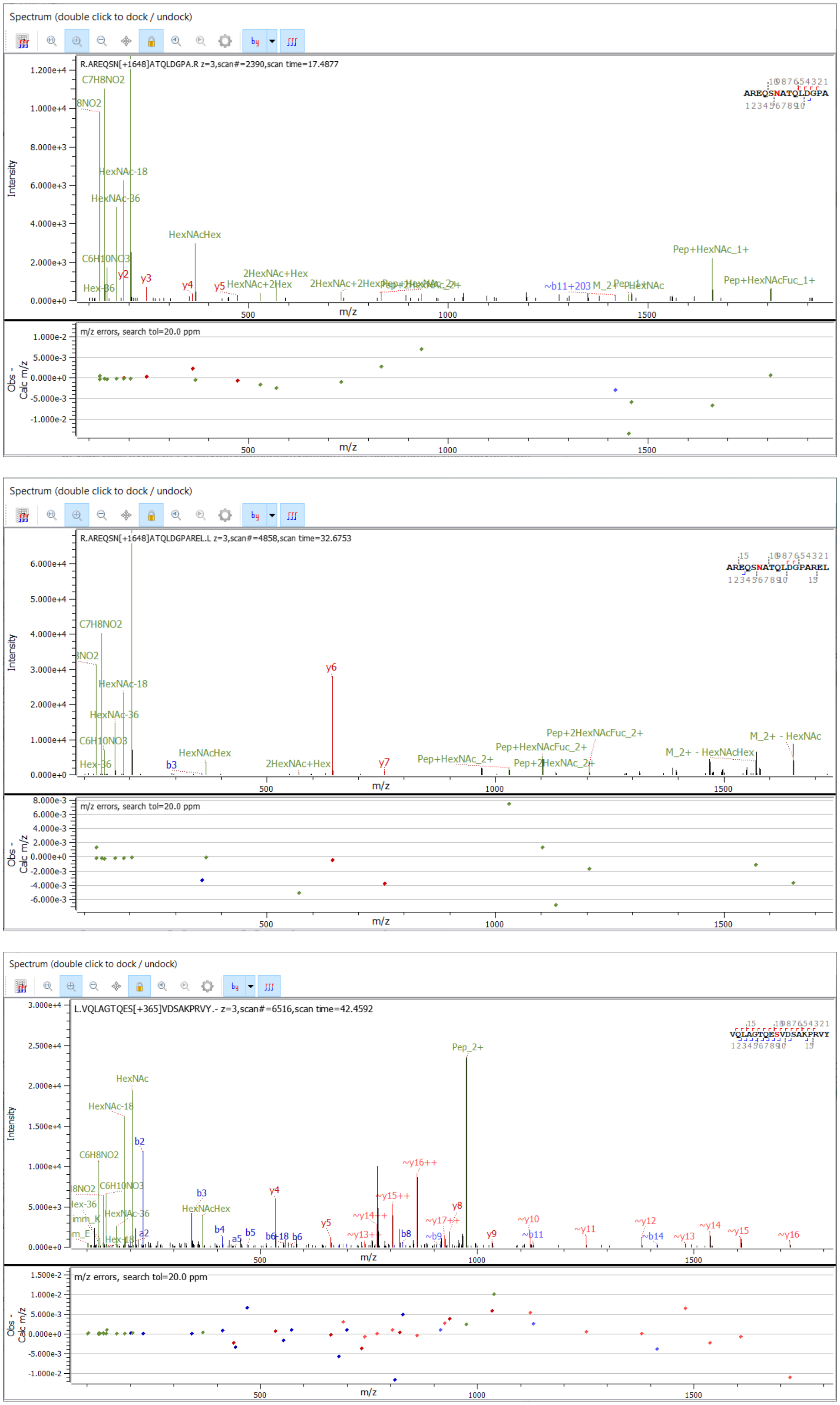

Figure S10. Vasopressin-neurophysin 2-copeptin peptides with N- and O-linked glycosylation.

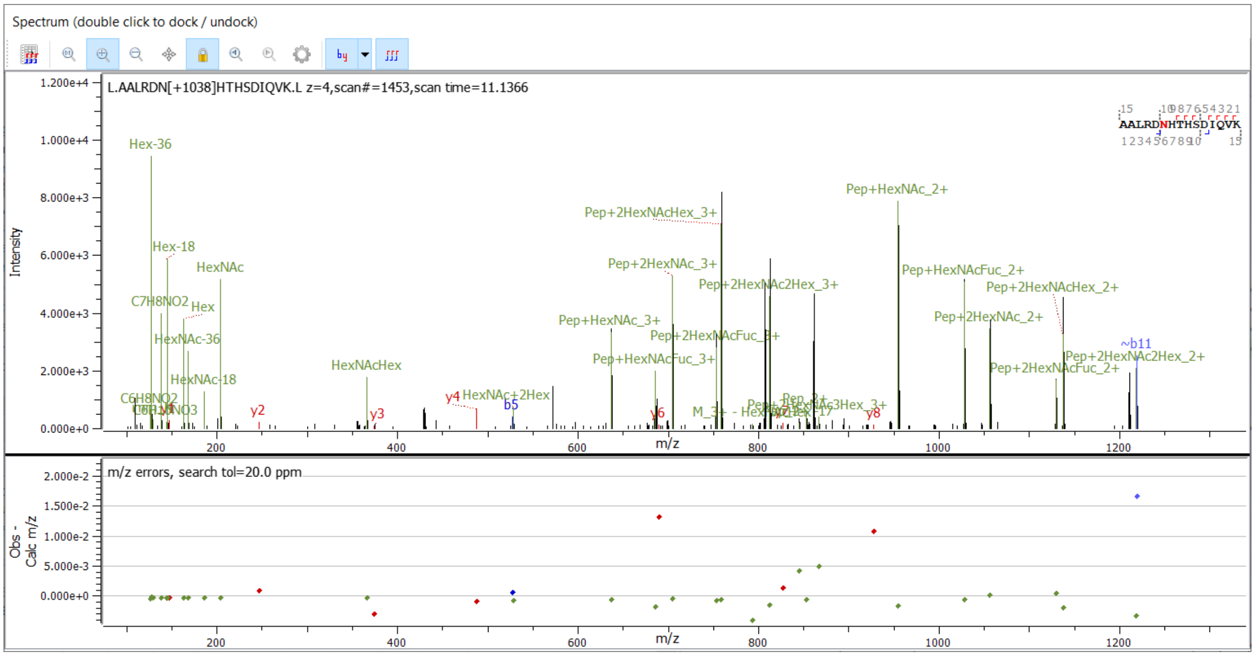

Figure S11. 5'-3' exonuclease PLD3 with N-linked glycosylation.

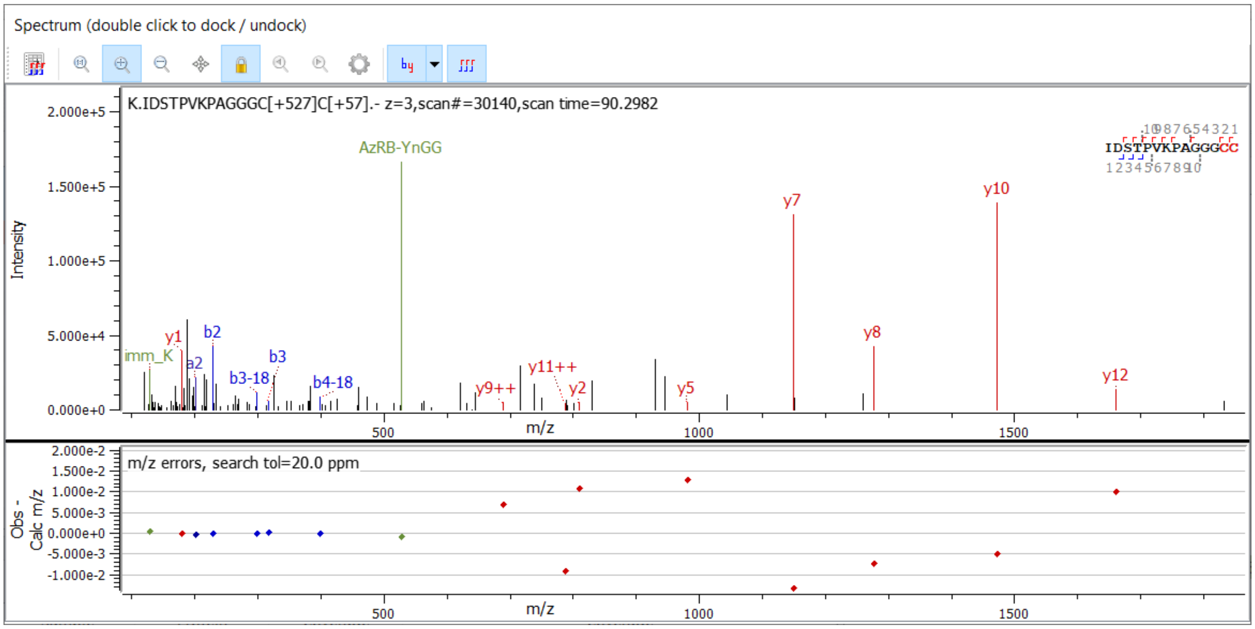

Figure S12. Scan 30140 in JMS133H.raw from PXD009155 matched to the C-terminal peptide from RAB1B_HUMAN (Ras-related protein Rab-1B) with AzRB-captured YnGG. Uniprot KB lists the second cysteine as a geranylgeranylation site. The prenylated peptides in S12 – S15 were not reported in the Supplemental to the Storck et al publication (PMC6544531).

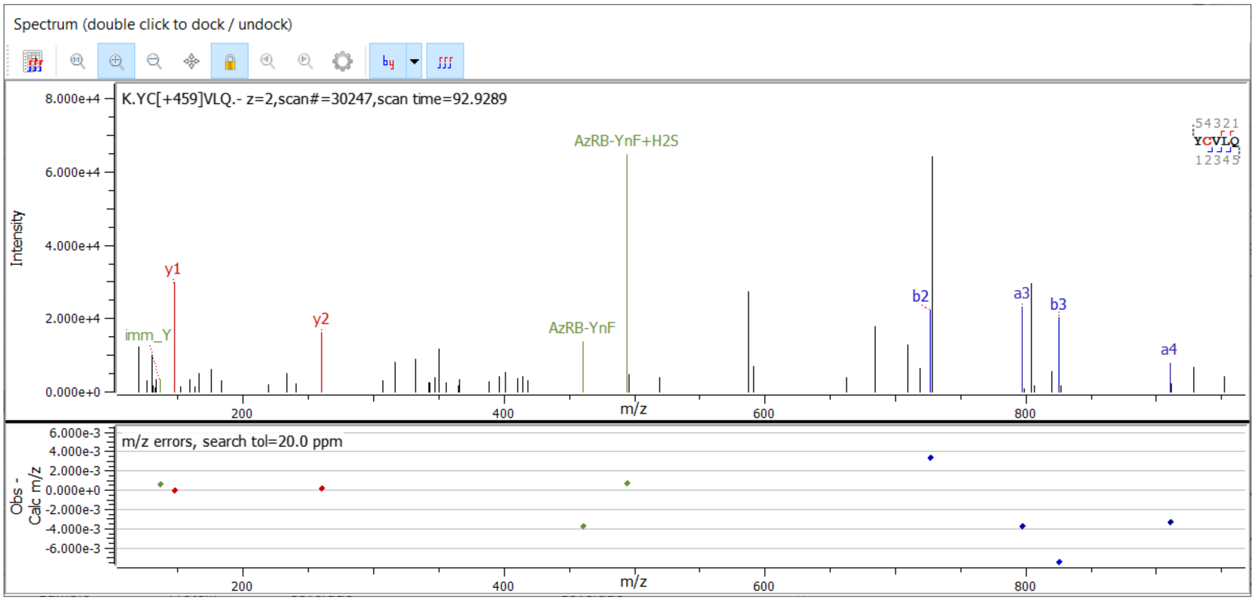

Figure S13. C-terminal peptide from UBP32_HUMAN (Ubiquitin carboxyl-terminal hydrolase 32) with AzRB-captured YnF (JMS133E.raw). Uniprot KB annotates this cysteine farnesylation site from "sequence analysis". As above, the protein of origin may be ambiguous for short peptide matches. Farnesylation, however, occurs near protein C-termini, usually with a Cxxx- motif, where – denotes protein C-terminus, which greatly limits the number of possible proteins.

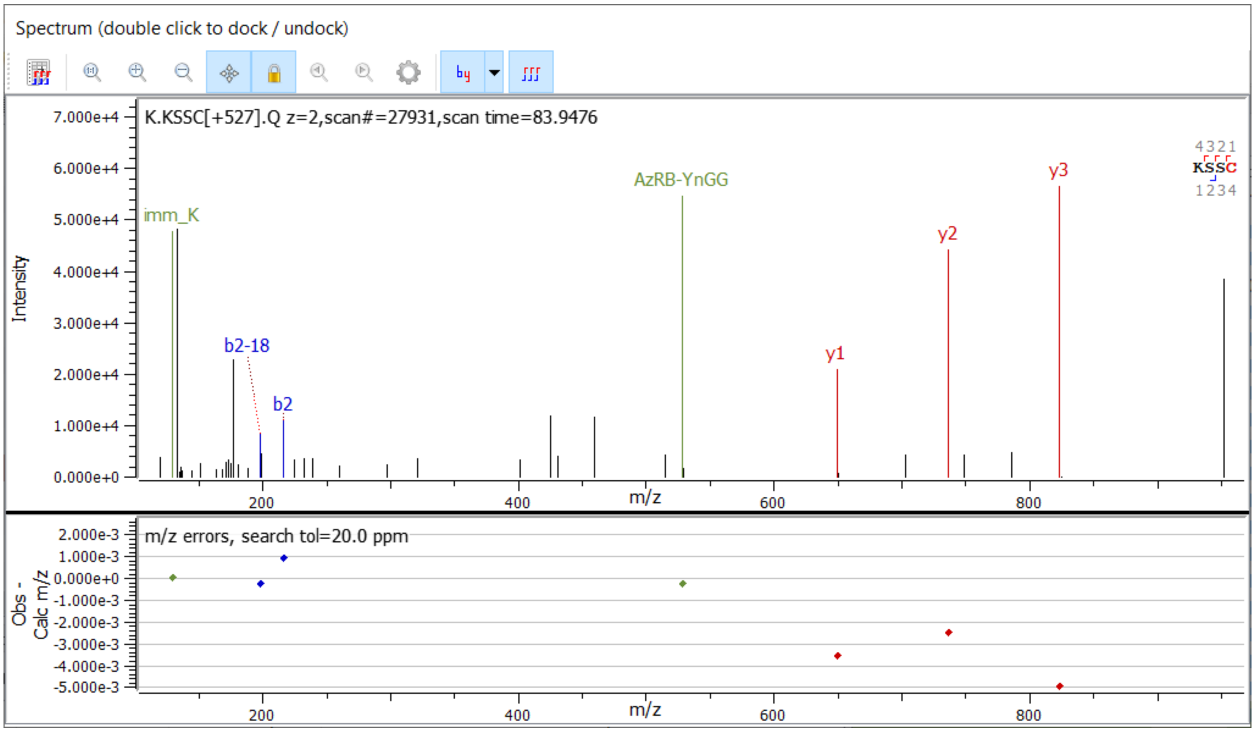

Figure S14. Peptide (C-terminal after processing) from RAP1B_HUMAN (Ras-related protein Rap-1b) with AzRB-captured YnGG (JMS133H.raw). Uniprot KB annotates this geranylgeranylation site. The unannotated peak at *m/z* 951.52 corresponds to a singly charged version of the peptide. Perhaps the precursor was a dimer?

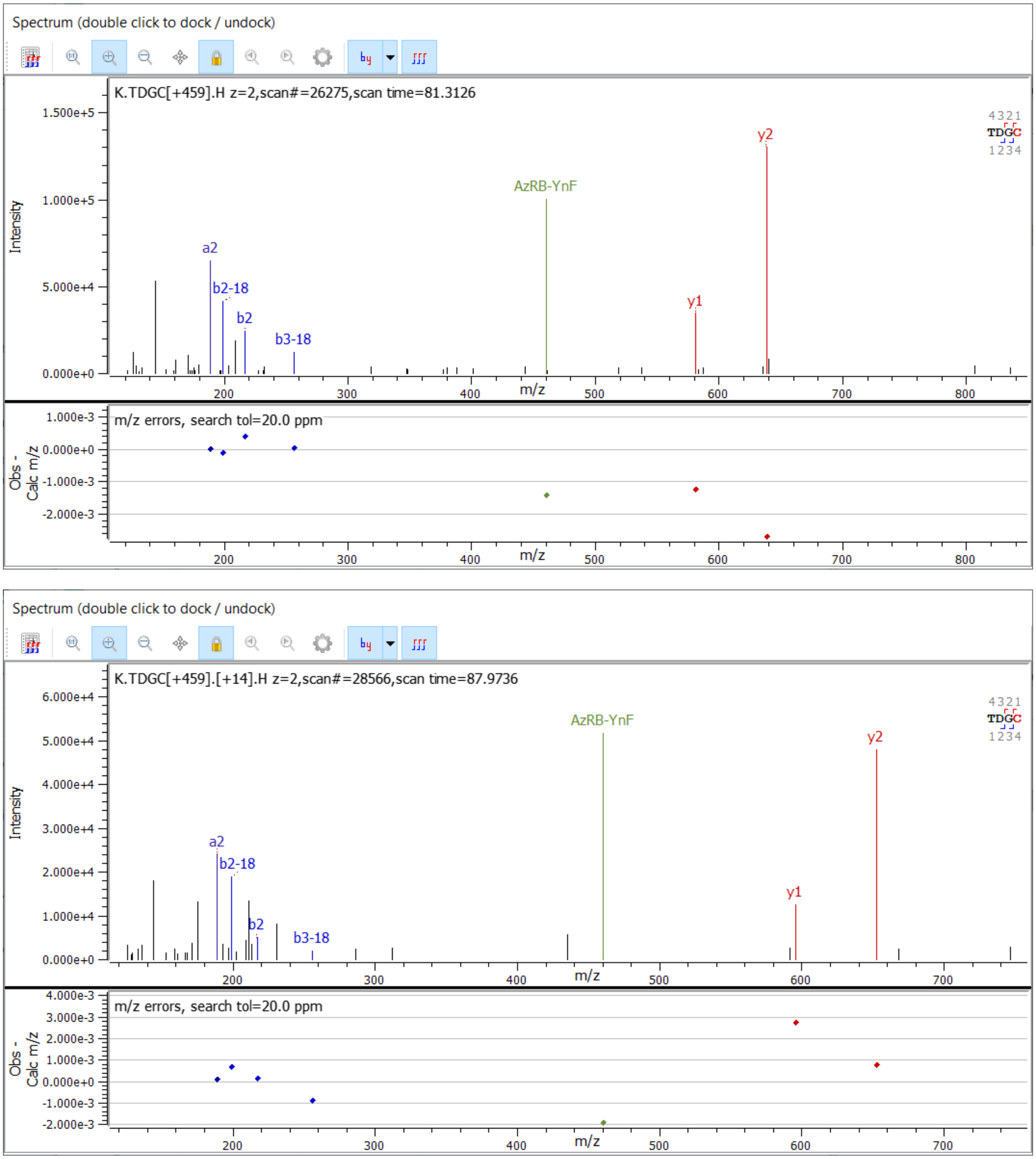

Figure S15. Spectra of a peptide with AzRB-captured YnF (JMS133E.raw) with and without C-terminal methylation. Byonic matched these spectra to CAP1_HUMAN, but this is probably not the protein of origin, because the peptide is not very close to the C-terminus of CAP1_HUMAN.

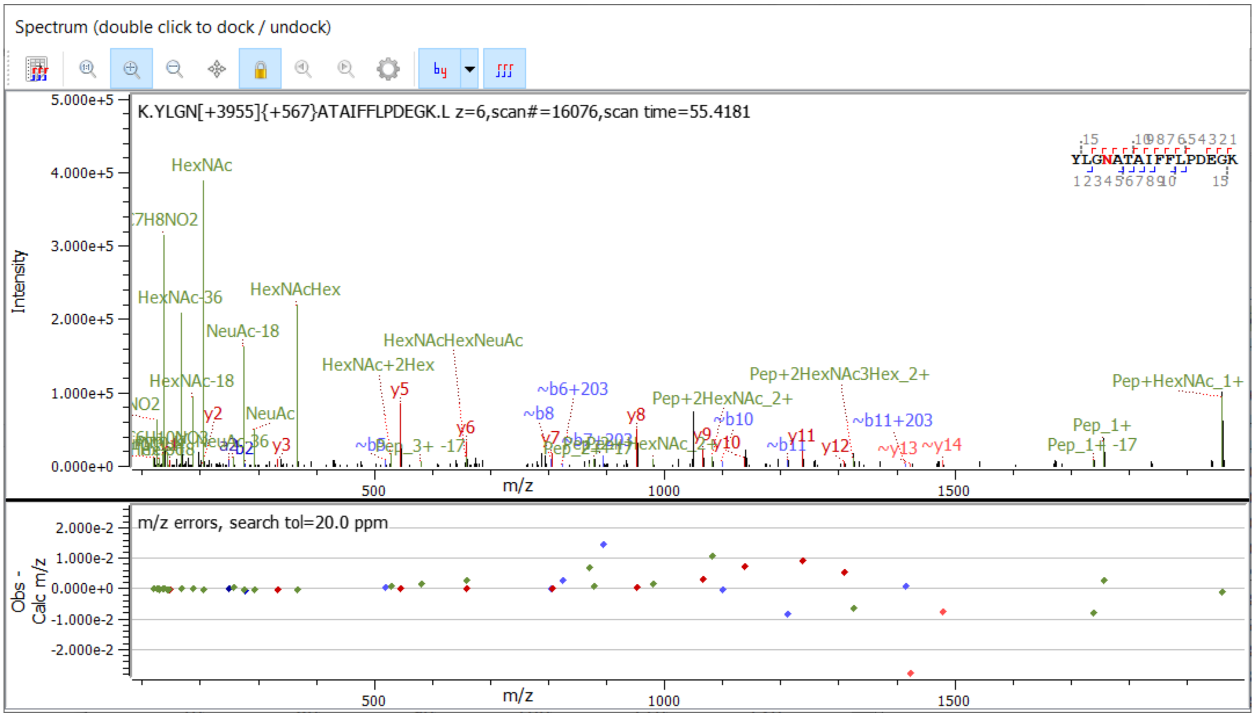

Figure S16. The peptide-spectrum match above is from a search of HUPO HGI data (file B_glycopepnew_HCDEThcDiTCIDpeptide.raw) that used MS2 Filtering Peaks to target a particular peptide in alpha-1-antitrypsin and Glycan Wildcard Search to find glycans not in the glycan database. The database glycan of mass 3955 Da is HexNAc(6)Hex(7)Fuc(3)NeuAc(4). The wildcard of mass 567 Da is probably HexNAc(2)Hex(1) with an off-by-one error in the monoisotopic mass call.

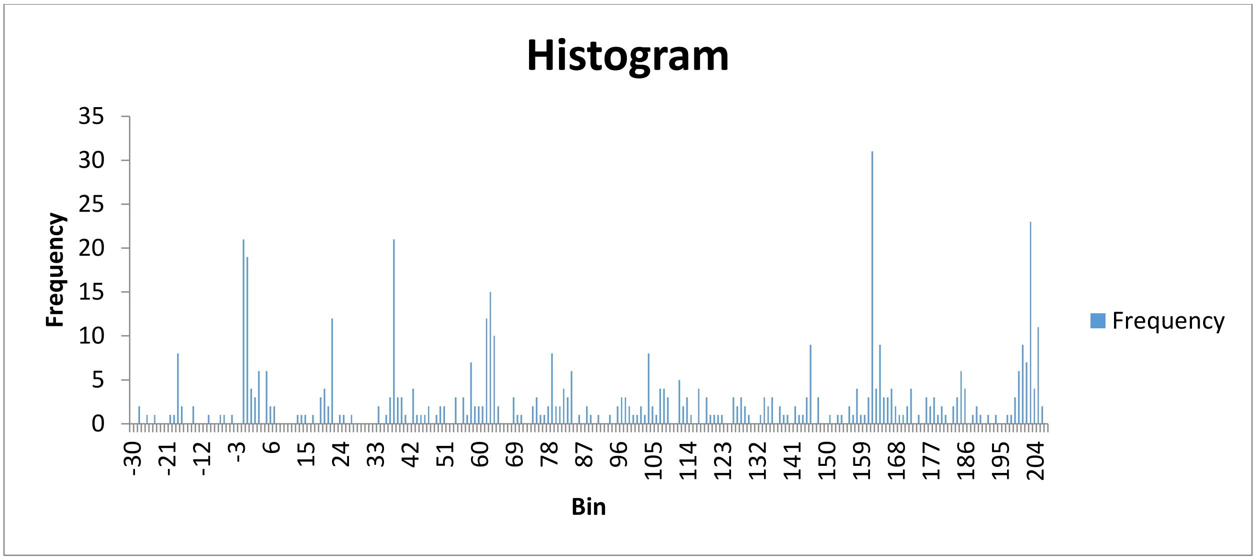

Figure S17. A histogram showing the masses of glycan wildcards on 547 high-scoring glycopeptide PSMs.

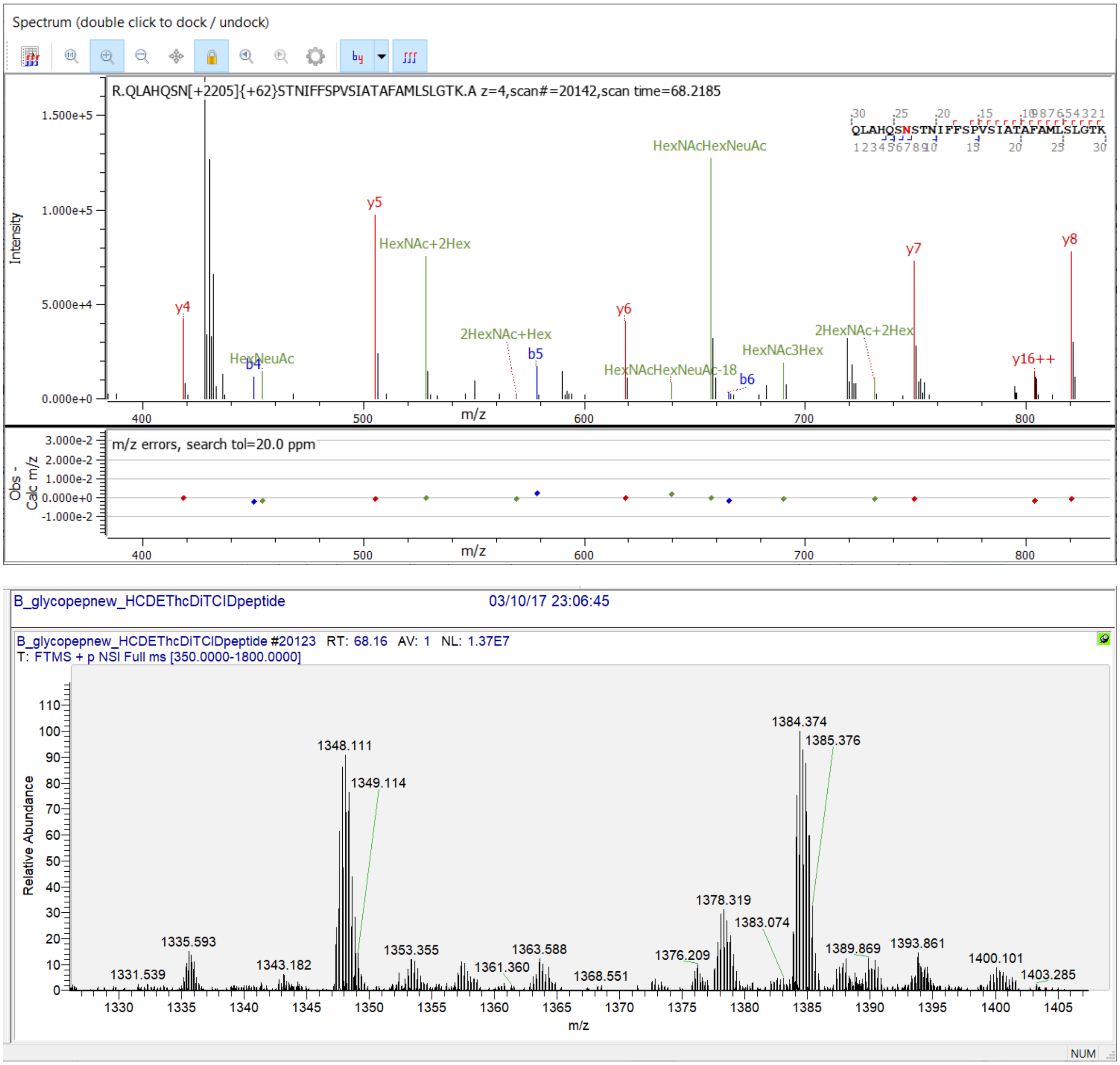

Figure S18. A glycan wildcard PSM with wildcard mass 61.916 Da. The database glycan of mass 2205 Da is HexNAc(4)Hex(5)NeuAc(2), one of the most common glycans in human plasma. We speculate that the wildcard represents Cu^{+} replacing a proton, which has mass delta 61.922 Da. The precursor isotope cluster for the MS2 spectrum is the *z*=4+ group labeled 1363.588. The much larger cluster at 1348.11 is the same glycopeptide without copper, and 1384.374 is the same peptide with HexNAc(4)Hex(5)Fuc(1)NeuAc(2).

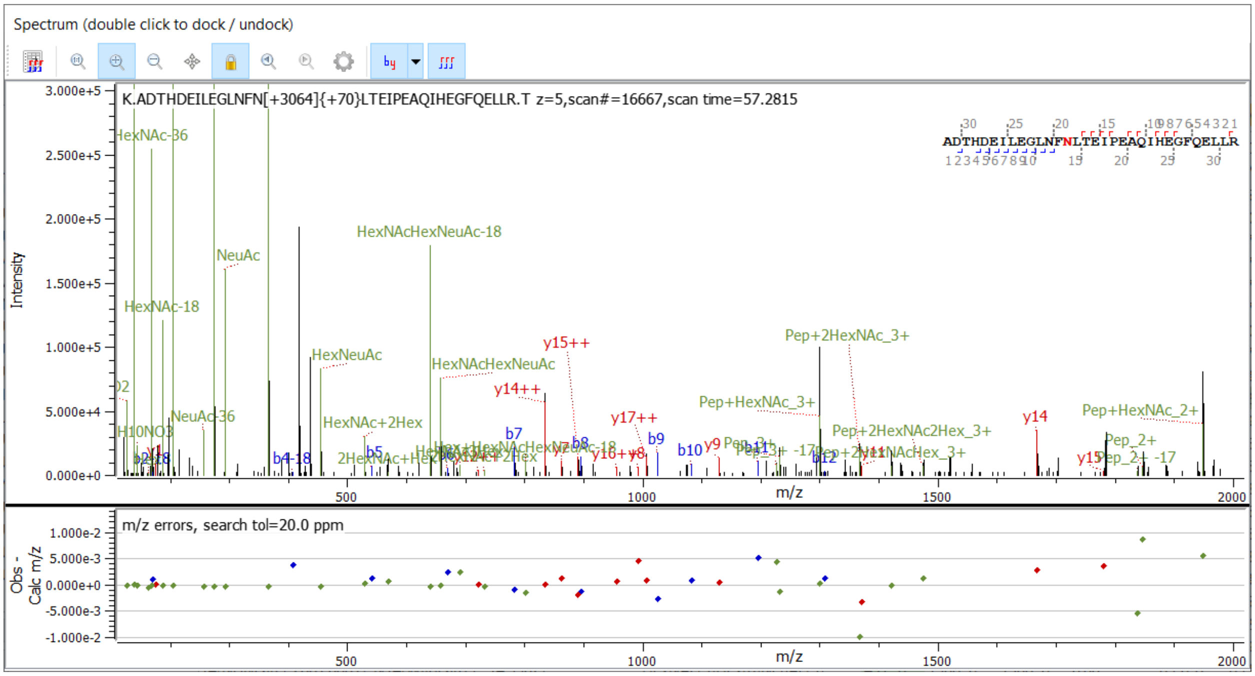

Figure S19. A glycan wildcard PSM. The database glycan of mass 3064 Da is HexNAc(6)Hex(6)NeuAc(3), but we do not have an explanation for the extra 70.056 Da.
